# Unprecedented “off‐pathway” [2+2] Cycloaddition‐Retroelectrocyclization Reaction Between an Unsymmetric Alkyne and Tetracyanoquinodimethane

**DOI:** 10.1002/anie.202506536

**Published:** 2025-04-17

**Authors:** Oscar Fernández‐Vera, Luca Sagresti, Luis M. Mateo, Tomas Torres, Giuseppe Brancato, Giovanni Bottari

**Affiliations:** ^1^ Departamento de Química Orgánica Universidad Autónoma de Madrid Campus de Cantoblanco Madrid 28049 Spain; ^2^ IMDEA‐Nanociencia c/Faraday 9 Campus de Cantoblanco Madrid 28049 Spain; ^3^ Institute for Advanced Research in Chemical Sciences (IAdChem) Universidad Autónoma de Madrid Madrid 28049 Spain; ^4^ Scuola Normale Superiore and CSGI Piazza dei Cavalieri 7 Pisa I‐56126 Italy; ^5^ Istituto Nazionale di Fisica Nucleare Largo Pontecorvo 3 Pisa I‐56100 Italy

**Keywords:** Anthryl‐fused derivative, Cyano‐Diels–Alder reaction, Cycloaddition‐retroelectrocyclization reaction, Regioselectivity, Tetracyanoquinodimethane

## Abstract

In recent years, the [2+2] cycloaddition‐retroelectrocyclization (CA‐RE) reaction between electron‐rich alkynes and electron‐deficient alkenes has emerged as one of the most effective synthetic routes to prepare a large variety of molecular and polymeric electron donor–acceptor systems. Besides its simplicity, fast rate, and high yield, this reaction may also display complete and predictable regioselectivity, as in the case when tetracyanoquinodimethane (TCNQ) is used in combination with unsymmetric, activated alkynes. Here, we report the first example of a [2+2] CA‐RE reaction between TCNQ and an aniline‐activated alkyne following an “inverted” regiochemistry, thus leading to the exclusive formation of an unexpected regioisomer in contrast to the expected one. A combined experimental and theoretical study helped us to unravel the peculiar reaction mechanism underlying the regioselectivity switching.

## Graphical abstract

The widely used [2+2] cycloaddition‐retroelectrocyclization reaction between tetracyanoquinodimethane (TCNQ) and an activated alkyne has been shown to occur with “inverted” regiochemistry to give exclusive formation of an unexpected regioisomer. A combined experimental and theoretical study helped unravel the peculiar reaction mechanism underlying the regioselectivity switching.

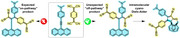

## Introduction

Molecular and polymeric push‐pull systems consisting of electron donor and acceptor moieties connected by a π‐conjugated spacer are highly investigated derivatives which have found multiple applications in many areas, including materials science, organic electronics, and photonics, owing to their interesting optoelectronic properties.^[^ ^1^ ^]^

For their preparation, one of the most straightforward and high‐yielding synthetic strategies is the [2+2] cycloaddition‐retroelectrocyclization (CA‐RE) “click” reaction between activated alkynes and electron‐deficient cyano‐substituted alkenes.^[^ ^2^, ^3^ ^]^

Within the large family of electron donor–acceptor (D–A) conjugates obtained following such a synthetic approach, those formed between activated alkynes and tetracyanoethylene (TCE), leading to molecular^[^ ^4^, ^5^ ^]^ and polymeric^[^ ^6^ ^]^ tetracyanobutadiene (TCBD)‐functionalized systems, are among the most abundant ones. For example, using this strategy D–A conjugates featuring the TCBD moiety linked to electro‐ and/or photoactive units such as C_60_ fullerene,^[^ ^7^ ^]^ corannulenes,^[^ ^8^ ^]^ truxenes,^[^ ^9^ ^]^ boron dipyrromethenes (BODIPYs),^[^ ^10^, ^11^ ^]^ porphyrins,^[^ ^12^, ^13^, ^14^ ^]^ subporphyrins,^[^ ^15^ ^]^ phthalocyanines,^[^ ^16^ ^]^ and subphthalocyanines^[^ ^17^, ^18^, ^19^, ^20^ ^]^ have been prepared. Moreover, in many of these D–A conjugates, photoinduced energy or electron transfer has been observed between the electron‐donor moiety and the strong electron‐acceptor TCBD.^[^ ^21^ ^]^ Besides its remarkable electron‐withdrawing features, the TCBD moiety also presents some interesting structural features resulting from the quasi‐orthogonal arrangement of its two dicyanovinyl (DCV) halves^[^ ^22^ ^]^ and the restricted rotation around the central C_2─_C_3_ bond. As a consequence, the formation of atropisomers has been observed and their separation achieved in a few cases.^[^ ^7^, ^15^, ^17^, ^20^ ^]^

**Scheme 1:**
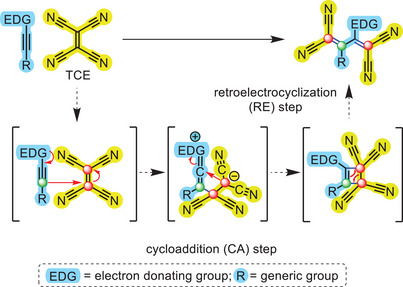
Two‐step mechanism for the [2+2] cycloaddition‐retroelectrocyclization (CA‐RE) reaction between an activated unsymmetric alkyne (blue) and tetracyanoethylene (TCE) (yellow). In the product as well as in the reaction intermediates (drawn within square brackets), the molecular fragments derived from the alkyne and the TCE moieties have been colored in blue and yellow, respectively. Newly formed bonds are colored in blue. Red arrows show electrons’ movement. For an easier identification of the structural rearrangement occurring throughout the CA‐RE reaction, in the product and intermediates the two “electrophilic” carbon atoms of the TCE double bond and the “nucleophilic” carbon atom of the alkyne moiety have been marked with a red and green dot, respectively.

Nowadays, it is widely accepted that the [2+2] CA‐RE reaction between activated alkynes and TCE occurs in two steps involving a zwitterionic intermediate that evolves towards a highly strained cyclobutene adduct, which has been isolated in a few cases (i.e., the CA step, Scheme 1).^[^ ^23^ ^]^ Upon a concerted rearrangement of the latter species, the TCBD‐functionalized D–A system is finally obtained (i.e., the RE step).

More recently, it has also been demonstrated that the TCBD‐based product has an autocatalytic effect on such reactions by promoting the generation of the cyclobutene intermediate, most likely through the formation of a charge‐transfer complex with the reactants.^[^ ^24^ ^]^

In an ongoing search for the preparation of D–A conjugates displaying improved electron‐accepting and optical absorption capability, especially in the visible region of the solar spectrum, 7,7,8,8‐tetracyanoquinodimethane (TCNQ) has been identified as an interesting alternative to TCE in [2+2] CA‐RE reactions.^[^ ^25^ ^]^

**Scheme 2:**
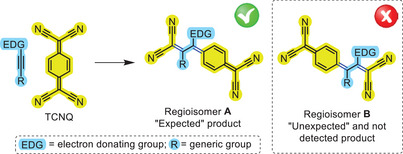
[2+2] Cycloaddition‐retroelectrocyclization (CA‐RE) reaction between an activated unsymmetric alkyne and TCNQ leading to “expected” *ext*TCNQ‐substituted regioisomer **A** as the only product. The “unexpected” and not detected *ext*TCNQ‐substituted regioisomer **B** has also been drawn within a dashed box. Newly formed bonds are colored in blue.

Using this strategy, a large number of D–A conjugates bearing an “extended” TCNQ moiety in their structure (hereafter referred to as *ext*TCNQ) have been prepared and studied.^[^ ^26^, ^27^, ^28^, ^29^ ^]^ In this context, it is worth noting that the [2+2] CA‐RE reaction between activated unsymmetric alkynes and TCNQ proceeds with total chemoselectivity (i.e., only the exocyclic double bond of TCNQ is involved in the reaction) and regioselectivity (i.e., only the regioisomer having the (dicyanomethylene)cyclohexa‐2,5‐diene fragment directly connected to the former C≡C carbon atom bearing the electron‐activating moiety is obtained, namely, regioisomer **A** in Scheme 2). Indeed, no formation of the alternative regioisomer (i.e., regioisomer **B** in Scheme 2) having the (dicyanomethylene)cyclohexa‐2,5‐diene and DCV fragments “swapped” with respect to **A** has ever been detected.

Herein, we report the first example of a [2+2] CA‐RE reaction between an aniline‐activated alkyne and TCNQ following an “inverted” regiochemistry, thus leading to the exclusive formation of an “unexpected” *ext*TCNQ regioisomer (i.e., regioisomer **B** in Scheme 2), as inferred by a wide range of spectroscopic and spectrometric techniques. Moreover, such “unexpected” regioisomer can be further transformed into an anthryl‐fused–*ext*TCNQ derivative by a thermally activated intramolecular cyano‐Diels–Alder (CDA) reaction, a process which could not occur in case the “expected” regioisomer was formed. Finally, quantum mechanical calculations were carried out to unravel the origin of such a “switched” regiochemistry, suggesting a novel “off‐pathway” mechanism involving a [6+6] cycloadduct intermediate.

## Results and Discussion

While many *ext*TCNQ‐functionalized D–A conjugates have been prepared by [2+2] CA‐RE reaction between unsymmetric alkynes and TCNQ, the origin of the complete regioselectivity observed in all previous studies was never fully elucidated.

A plausible rationale of such a regioselectivity can be put forward attending to some mechanistic considerations. In principle, two reaction pathways can be postulated for this reaction, namely an “on‐pathway” and an “off‐pathway” mechanism (Scheme S2.1). Both pathways involve the attack of the more nucleophilic alkyne carbon atom (i.e., the one further away with respect to the electron‐donating group) to the TCNQ exocyclic double bond, thus leading to the formation of two zwitterionic intermediates. However, the “on‐pathway” mechanism leading to the observed regioisomer **A** is preferred over the “off‐pathway” one leading to the undetected regioisomer **B**. In the former case, in fact, the attack of the more nucleophilic alkyne carbon atom takes place at the more electrophilic carbon atom of the C═C DVC unit (i.e., the one bearing the two cyano groups) and a gain of aromaticity is obtained in one of the CS reaction intermediates, in contrast to the “off‐pathway” mechanism (Scheme S2.1).

**Scheme 3:**
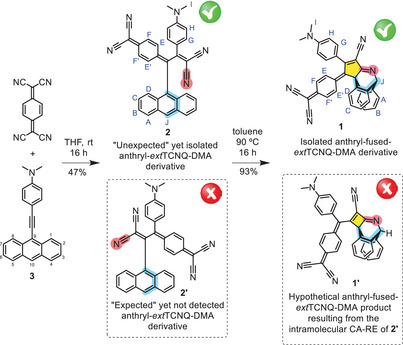
Synthetic route to “unexpected” anthryl–*ext*TCNQ–DMA **2** and its CDA product anthryl‐fused–*ext*TCNQ–DMA **1**. The structures of the “expected” yet not detected anthryl–*ext*TCNQ–DMA derivative **2′** and its hypothetical anthryl‐fused CDA adduct **1′** have been drawn within a dashed box. In **2** and **2′**, the cyano “dienophile” on the dicyanovinylene fragment and the anthryl “diene” unit have been colored in red and blue, respectively. The same color code has been used to identify the former “dienophile” and “diene” in the anthryl‐fused CDA adducts **1** and **1′**. The five‐ and four‐member rings in **1** and **1′**, respectively, have been filled in orange color. The numbering of the anthryl moiety positions is presented for derivative **3**.

Based on this interpretation, we carried out the reaction between TCNQ and 4‐(anthracen‐9‐ylethynyl)‐DMA **3**, an activated alkyne previously combined with TCBD,^[^ ^30^ ^]^ expecting the formation of regioisomer **2′** (Scheme 3). From such reaction, a single product was isolated having a molecular mass of 525.2 *m*/*z*, that is the sum of the molecular mass of **3** and TCNQ (Figure S3.3).

The ^1^H‐NMR spectrum of the isolated product further supported this assumption showing peaks with integral, multiplicity, and chemical shifts consistent with the molecular structure of **2′** (Figure S3.1).

**Figure 1:**
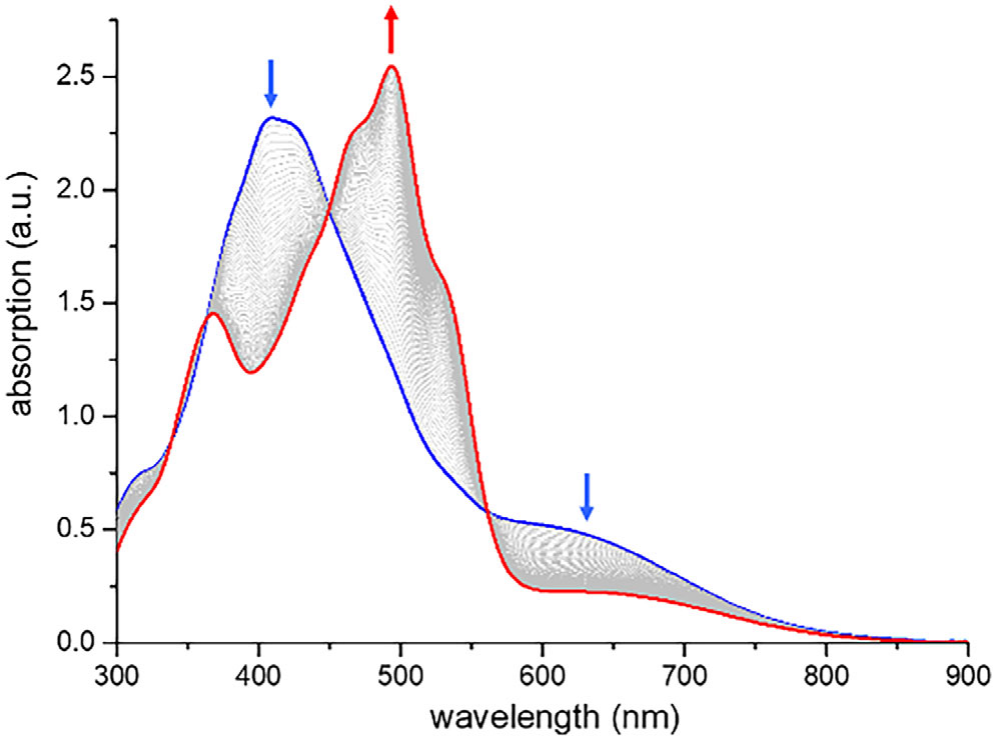
UV–vis absorption spectra of a toluene solution of anthryl–*ext*TCNQ–aniline **2** heated at 90 °C recorded at *t* = 0 (blue line) and *t* = 120 min (red line); intermediate spectra recorded every 2 min (grey lines). The blue and red line spectra correspond to that of **2** and **1**, respectively.

Next, the isolated product was subject to thermal treatment. Interestingly, heating a toluene solution of the anthryl–*ext*TCNQ–DMA derivative at 90 °C over 2 h promoted its transformation into a new product as documented by UV–vis absorption spectroscopy (Figure 1). Furthermore, the presence of several isosbestic points at 338, 363, 450, and 561 nm suggested a direct, compound‐to‐compound thermally induced transformation taking place without the formation of any intermediate species.

Silica gel column chromatography was successfully employed to isolate the product resulting from the thermally induced transformation of anthryl–*ext*TCNQ–DMA. Mass spectrometry of the isolated species showed that the new compound has a molecular mass identical to that of its precursor (Figure S3.12), thus suggesting that the thermally induced transformation only triggers a rearrangement of the atoms’ connectivity in the anthryl–*ext*TCNQ–DMA precursor.

**Figure 2:**
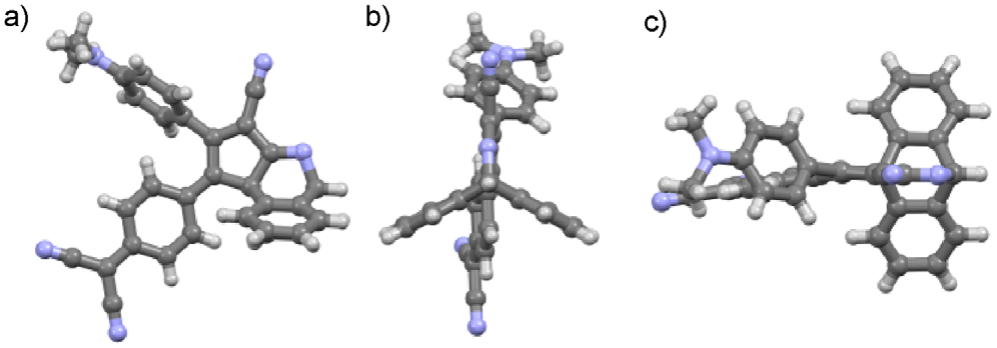
a) Side, b) front, and c) top view (with respect to the cyclopentene five‐membered ring) of the X‐ray crystal structure of **1**. Carbon atoms are colored in light gray, nitrogen atoms in light blue, and hydrogen atoms in white. Chloroform molecules of crystallization have been omitted for clarity. Structure details are given in the Supporting Information.

Dark‐red colored single crystals of the isolated product of the thermally induced transformation of anthryl–*ext*TCNQ–DMA suitable for X‐ray diffraction analysis were obtained by slow evaporation of a chloroform solution of the compound. X‐ray structural analysis of such crystals allowed to unequivocally determine the compound's molecular structure (Figure 2), which corresponds to that of anthryl‐fused–*ext*TCNQ–DMA **1** (Scheme 3).

Compound **1** presents a bicyclic structure in which the two “outer” benzene rings of the former anthryl moiety are arranged so as to form a ∼120° angle between them and, in turn, with the newly formed five‐member ring (Figure 2b). The formation of such species could be explained considering an intramolecular CDA reaction between a cyano moiety of the DCV unit and the central benzene ring of the anthryl moiety, as previously observed for an anthryl‐fused–TCBD–DMA derivative.^[^ ^30^ ^]^

Once the structure of **1** was unequivocally determined, considering that it could only be formed via an intramolecular CDA reaction, we were in the position, using a “retrosynthetic” approach, to validate the hypothesized structure of its “open” anthryl–*ext*TCNQ–DMA precursor, whose X‐ray crystal structure could not be obtained. Surprisingly, such analysis pointed out that derivative **2′**, which we initially thought was the product isolated from the reaction of **3** and TCNQ, could not give rise to **1** by intramolecular CDA reaction. Derivative **2′** presents an inadequate relative arrangement of the cyano “dienophile” and the anthryl “diene” (marked in red and blue, respectively, in Scheme 3), which would lead, in the case of a hypothetical intramolecular CDA reaction, to **1′**, a bicyclic compound presenting a highly strained, cyclobutene‐type ring (colored in orange in Scheme 3).

On the other hand, the retrosynthetic analysis of **1** clearly points out that the “unexpected” derivative **2** has to be the precursor of **1**. In the former compound, the presence of the DCV fragment “conjugated” to the DMA moiety would allow an optimal relative arrangement of the cyano “dienophile” and the anthryl “diene” (marked in red and blue, respectively, in Scheme 3) for the intramolecular CDA to take place leading to **1**.

**Figure 3:**
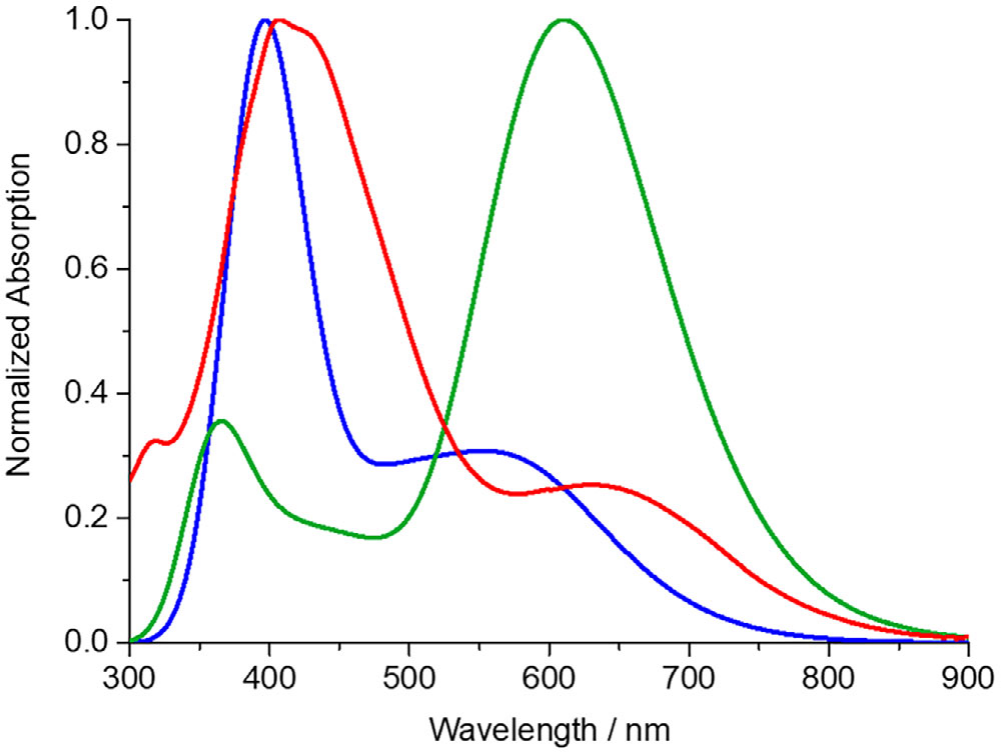
Observed UV–vis absorption spectrum in THF of “unexpected” derivative **2** (blue line), and calculated UV–vis absorption spectra in THF of “unexpected” derivative **2** (red line) and “expected” derivative **2′** (green line).

To further confirm the structure of **2**, we turned our attention to UV–vis absorption spectroscopy. The UV–vis absorption spectrum of **2** in THF is dominated by an intense high‐energy band peaked at around 400 nm and a significantly weaker band in the low‐energy region between 550 and 900 nm (Figure 3, blue line). Interestingly, the former band lies in a spectral region where many DMA–TCBD‐based derivatives also show a strong absorption resulting from the charge transfer between the electron‐donating DMA and its conjugated electron‐accepting DCV moiety.^[^ ^21^ ^]^ The UV–vis absorption spectrum of **2** in THF was also calculated showing a good agreement with the experimental spectrum (Figure 3, red line).

On the other hand, **2** shows no sign of any intense band between 600 and 900 nm, a typical “fingerprint” seen in all the “expected” DMA–*ext*TCNQ derivatives reported to date, which stems from the charge transfer between the DMA and its conjugated electron‐accepting (DCV)cyclohexa‐2,5‐diene fragment.^[^ ^27^, ^29^ ^]^ In this context, it is interesting to notice that the calculated UV–vis absorption spectrum of the “expected” yet not isolated compound **2′** in THF does also reproduce such intense band in the low‐energy spectral region (Figure 3, green line). These results from UV–vis absorption and theoretical calculations further support the notion of the proposed structure of **2** as that of the “unexpected” anthryl–*ext*TCNQ–DMA regioisomer obtained.

**Figure 4:**
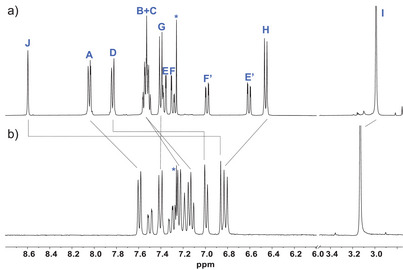
^1^H‐NMR spectra (CDCl_3_) of a) “unexpected” anthryl–*ext*TCNQ–DMA **2** and b) anthryl‐fused–*ext*TCNQ–DMA **1**. Capital letters refer to protons’ assignment of **1** and **2** in Scheme 2. * Denotes residual solvent signals.

Once determined, the structure of **1** and its “unexpected” precursor **2**, their ^1^H‐NMR spectra were revised in light of their structural features (Figure 4). A spectral comparison of the two compounds shows that all the aromatic hydrogens in “fused” **1** experience an upfield shift compared to those of its “open” analog **2**, except for H_G_ and H_H_ which move downfield. Among the aromatic protons, the most pronounced shift (i.e., ∼1.9 ppm) is observed for H_J_ due to a change in the hybridization of the carbon atom it is attached to, that is, from sp^2^ in **2** to sp^3^ in **1**, and the “loss” of the aromatic character of the former central anthryl ring.

**Figure 5:**
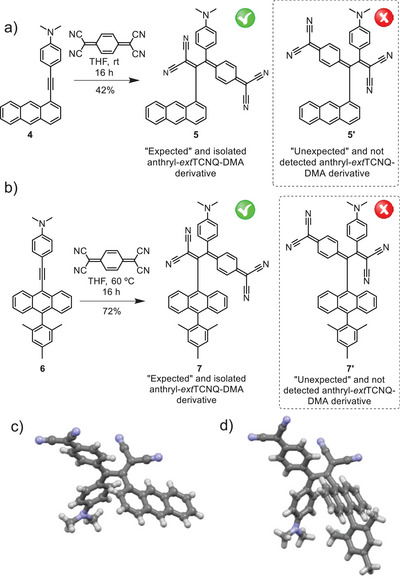
Synthetic route to “expected” and isolated anthryl–*ext*TCNQ–DMA a) **5** and b) **7**, and “unexpected” and not detected anthryl–*ext*TCNQ–DMA a) **5′** and b) **7′** (drawn within a dashed box). X‐ray crystal structure of “expected” anthryl–*ext*TCNQ–DMA c) **5** and d) **7**. Carbon atoms are colored in light grey, nitrogen atoms in light blue, and hydrogen atoms in white. Chloroform molecules of crystallization have been omitted for clarity. Structure details are given in the Supporting Information.

With the aim of rationalizing the “unexpected” formation of **2** in the [2+2] CA‐RE reaction between **3** and TCNQ, two control reactions were carried out. 4‐(Anthracen‐1‐ylethynyl)‐DMA **4**, a regioisomer of **3** with the ethynyl unit attached at the anthryl position 1, was prepared and reacted with TCNQ in a [2+2] CA‐RE reaction (Figure 5a). In the same experimental conditions leading to the formation of **2**, the reaction afforded a single product, which was isolated. Interestingly, structural determination of such species by single‐crystal X‐ray diffraction analysis showed that it corresponds to the “expected” anthryl–*ext*TCNQ–DMA **5** (Figure 5c). Conversely, no sign of “unexpected” compound **5′** formation was observed (Figure 5a).

In another control experiment, a [2+2] CA‐RE reaction between TCNQ and 4‐((10‐mesitylanthracen‐9‐yl)ethynyl)‐DMA **6**, a compound formally obtained by replacing the hydrogen at the anthryl 10 position of **3** by a mesityl unit, was investigated (Figure 5b). Also in this case, high‐quality single crystals of the isolated product suitable for X‐ray determination were obtained showing that it corresponds to “expected” anthryl–*ext*TCNQ–DMA **7** (Figure 5d). Also in this case, no formation of the “unexpected” product **7′** was detected (Figure 5b). Further supporting the formation of the “expected” regioisomers **5** and **7** is the presence in their UV–vis absorption spectra of the characteristic intense, low‐energy absorption band ascribable to the DMA‐(DCV)cyclohexa‐2,5‐diene charge transfer transition (Figure S4.1). It is worth noticing that a thermal treatment of the “expected” derivatives **5** and **7** in toluene did not result, as expected, in their transformation into any new species, suggesting that the arrangement of the cyano “dienophile” of the DCV fragment and the anthryl “diene” in both derivatives precludes the formation of any intramolecular CDA product.

In order to shed some light on the “unexpected” formation of **2**, quantum mechanical calculations at the density functional theory (DFT) level were carried out. With this aim, the “on‐pathway” and “off‐pathway” [2+2] CA‐RE reaction profiles of **3**, **4**, and **6** with TCNQ were investigated. In all cases, results showed that the reaction rate‐determining step (i.e., the formation of the first intermediate from a pre‐reaction complex between the DMA‐based moiety and TCNQ, see Figures S6.1–S6.3) along the “on‐pathway” reaction is characterized by a significantly lower energy barrier (i.e., TS1) than the corresponding “off‐pathway” route by about 15 kcal mol^−1^, despite the “unexpected” product appeared somewhat more stable (i.e., ∼1–3 kcal mol^−1^) than the “expected” one (see Figures S6.1–S6.3).

**Figure 6:**
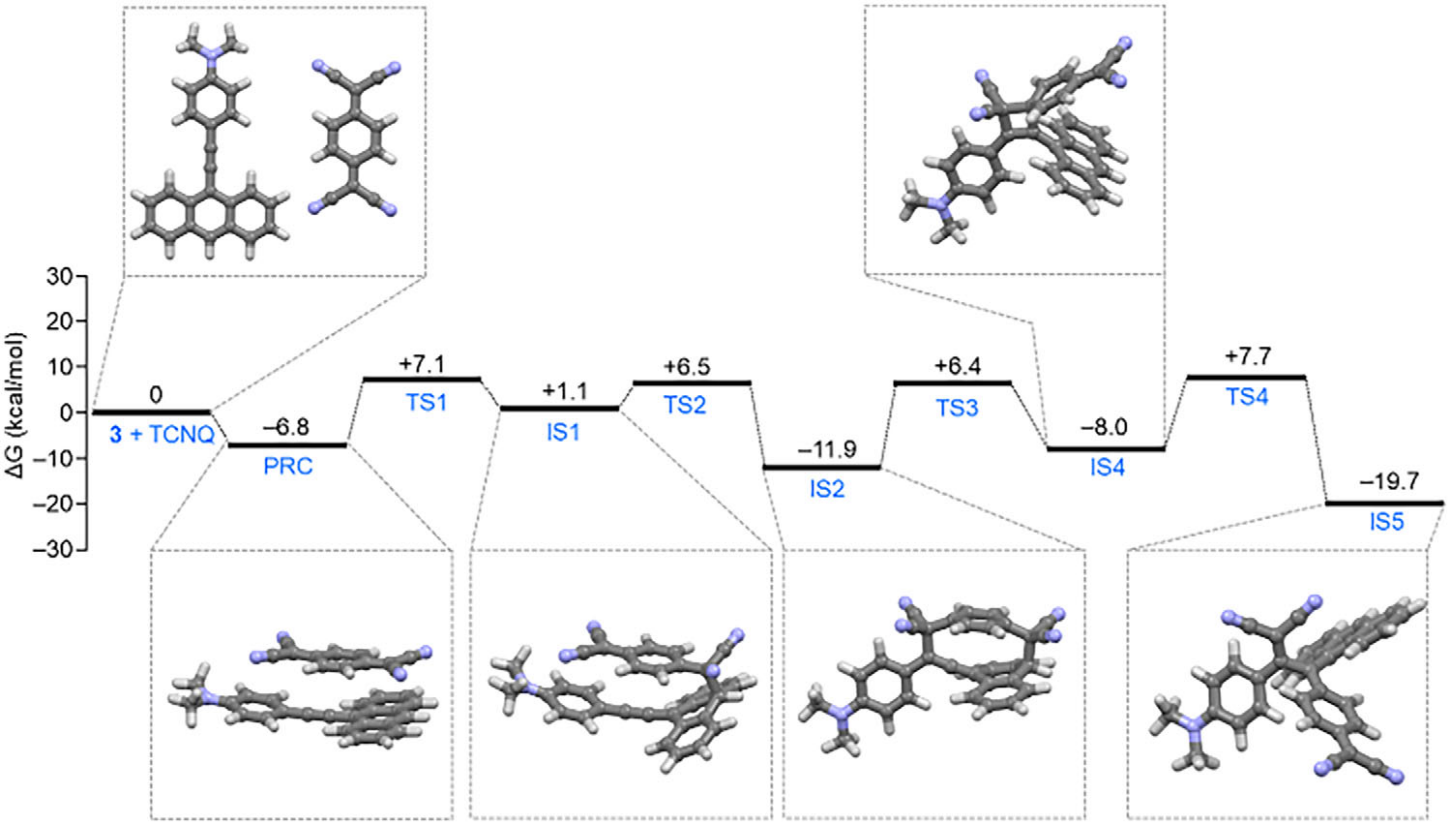
DFT‐calculated reaction coordinates between **3** and TCNQ leading to the formation of “unexpected” derivative **2** through the formation of a [6+6] cycloadduct intermediate (i.e., IS2) followed by a rearrangement. The calculated structures of the two reactants, the pre‐reaction complex (i.e., PRC), and the intermediates (i.e., IS) have also been represented and the respective energy levels included. The energy levels of the transition states (i.e., TS) have also been included.

Hence, DFT calculations confirmed that the “on‐pathway” reaction leading to the “expected” products **5** and **7** resulted more favourable from the kinetic viewpoint.^[^ ^31^ ^]^ Yet, the formation of the “unexpected” regioisomer **2** remained elusive in light of the present analysis. Such a puzzling observation prompted us to investigate further the [2+2] CA‐RE reaction between **3** and TCNQ, seeking alternative reactive pathways with respect to those illustrated in Scheme S2.1. Interestingly, we discovered another viable reaction mechanism that fully supported the formation of conjugate **2** in place of **2′** (Figures 6 and S6.4).

**Scheme 4:**
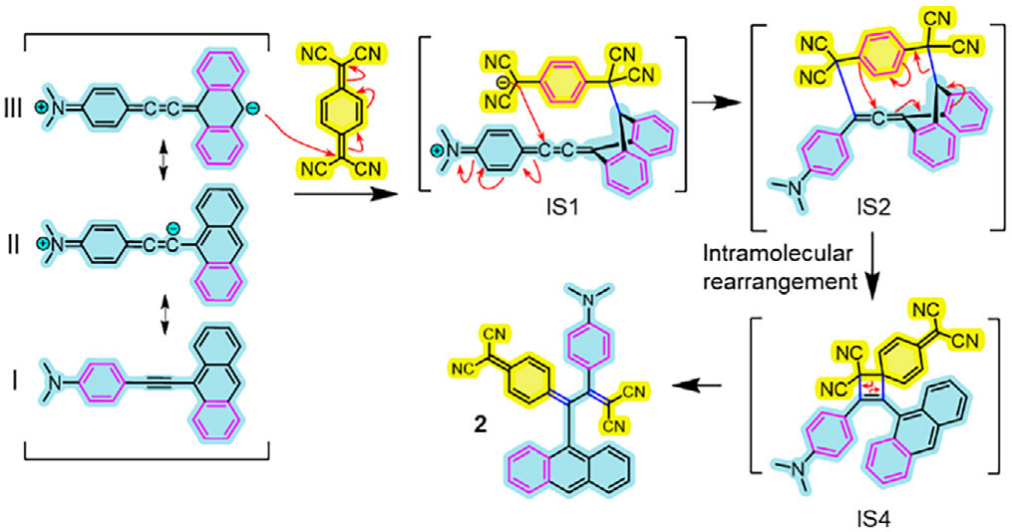
Proposed mechanism for the formation of the “unexpected” anthryl–*ext*TCNQ–DMA **2** resulting from the reaction between 4‐(anthracen‐9‐ylethynyl)‐DMA **3** and TCNQ. In **2** as well as in the reaction intermediate species IS1, IS2, and IS4, the molecular fragments derived from the alkyne and the TCNQ moieties have been inner colored in blue and yellow, respectively. Red arrows show attacking positions. In the case of reactant **3**, three of its possible resonance forms (i.e., I, II, and III) have been drawn. Purple‐colored six‐member rings indicate Clar's sextets.

The reaction starts with the attack of the anthryl carbon atom in position 10 of **3** (i.e., C10 in Figure S6.5) to the exocyclic alkene carbon atom of the TCNQ moiety (Figures 6 and S6.4). Compared to the previously investigated “on‐pathway” reaction, such initial reaction step is characterized by a significantly lower energy barrier (i.e., 7.1 kcal mol^−1^) and a more stable IS1 intermediate (i.e., ∼4 kcal mol^−1^) (Figures S6.1 and S6.4). At this point, it is worth noting that the marked nucleophilic character of C10 in **3**, as compared to both alkyne carbon atoms C11 and C12, was also supported by the reactive site analysis carried out in terms of the condensed Fukui functions, which shows a similar *f_i_^−^* parameter (i.e., the nucleophilic descriptor) with respect to the latter (Figure S6.5). Then, the reaction proceeds through the formation of a formal [6+6] cycloadduct intermediate (i.e., IS2) characterized by a rather stable energy (∼11 kcal mol^−1^) owing to the simultaneous presence of 4 aromatic six‐member rings (Scheme 4). This intermediate undergoes a rearrangement leading to intermediate IS4 and then to **2** (Figures 6 and S6.4), thus following the same remaining route seen in the previous “off‐pathway” of Figure S6.1b. Notably, the “off‐pathway” reaction profile proposed here (as depicted in Scheme 4) shows a more favorable reaction mechanism than the “expected” one from both the kinetic and thermodynamic standpoints, thus nicely supporting the formation of the “unexpected” regioisomer **2**. Note that the latter mechanism seems peculiar to compound **3** and not feasible to **4** and **6** for either electronic or geometric consideration (for precursor **4**, the corresponding condensed Fukui function of C10 showed a poor nucleophilic character, as shown in Figure S6.6, while in **6** the same carbon atom is not a suitable nucleophile since it is sterically hindered).

## Conclusions

In this study, we report the first example of a [2+2] CA‐RE reaction between TCNQ and an asymmetric electron‐rich alkyne following an “inverted” regiochemistry with respect to all previously reported syntheses of this kind. Interestingly, by applying a thermal treatment, the “unexpected” regioisomer was further converted into a bicyclic, anthryl‐fused–*ext*TCNQ derivative through an intramolecular CDA reaction. Theoretical calculations, in combination with some control experiments, highlighted the existence of an alternative mechanism involving a [6+6] intermediate (i.e., “off‐pathway”) in contrast to the usual “on‐pathway,” thus providing a molecular understanding of the key features promoting a regioselectivity switch in the CA‐RE reaction. We reckon that this work adds a valuable contribution to the [2+2] CA‐RE reaction between TCNQ and asymmetric alkynes by shedding light on the intricate mechanistic details and regioselective control that govern its outcome. The discovery of an “off‐pathway” mechanism opens new avenues for the design of novel D–A systems with tailored electronic properties.

## Conflict of Interests

The authors declare no conflict of interest.

## Supporting information



Supplementing information

## Data Availability

The data that support the findings of this study are available in the Supporting Information of this article.
