# Supplementary material for: Unprecedented “off‐pathway” [2+2] Cycloaddition‐Retroelectrocyclization Reaction Between an Unsymmetric Alkyne and Tetracyanoquinodimethane
Source: Angew Chem Int Ed Engl. 2025 Apr 17;64(24):e202506536. doi: 10.1002/anie.202506536 (PMC12144899; doi:10.1002/anie.202506536)
Supplement: Supplementary file 1 — Supplementing information [file ANIE-64-e202506536-s001.pdf]

Supporting Information for

Unprecedented "Off-Pathway" [2+2] Cycloaddition-Retroelectrocyclization Reaction between an Unsymmetric Alkyne and Tetracyanoquinodimethane

Oscar Fernández-Vera,[a] Luca Sagresti,[d] Luis M. Mateo,[a,b] Tomas Torres,*[a,b,c] Giuseppe Brancato,*[d] and Giovanni Bottari*[a,b,c]

a Departamento de Química Orgánica, Universidad Autónoma de Madrid, Campus de Cantoblanco, 28049 Madrid (Spain)

b IMDEA-Nanociencia, Campus de Cantoblanco, 28049 Madrid (Spain)

c Institute for Advanced Research in Chemical Sciences (IAdChem), Universidad Autónoma de Madrid, 28049, Madrid (Spain)

d Scuola Normale Superiore and CSGI, Piazza dei Cavalieri 7, 56126 Pisa (Italy) and Istituto Nazionale di Fisica Nucleare, Largo Pontecorvo 3, 56100 Pisa (Italy)

E-mail: giovanni.bottari@uam.es; giuseppe.brancato@sns.it; tomas.torres@uam.es.

<u>**Table of Content**</u>

		Page
1.	Materials and methods………………………………………………………	S-2
2.	Mechanistic insight on the [2+2] cycloaddition-retroelectrocyclization (CA-RE) reaction between a generic, activated unsymmetric alkyne and TCNQ…..	S-6
3.	Synthesis and characterization of anthryl-fused−*ex*TCNQ−DMA 1 and anthryl−*ex*TCNQ−DMA derivatives 5 and 7…………………………………	S-7
4.	UV-vis absorption spectra of anthryl-fused–*ext*TCNQ–DMA 1 and anthryl−*ext*TCNQ−DMA 2, 5 and 7…………………………………………...	S-37
5.	Electrochemical characterization of anthryl-fused−*ext*TCNQ−DMA 1 and anthryl−*ext*TCNQ−DMA 2……………………………………………......	S-39
6.	Quantum mechanical calculations studies ……………………………………	S-41
7.	Supporting Information References………………………………………	S-46

Abbreviations:

APCI = atmospheric-pressure chemical ionization; CFF = condensed Fukui function; CA-RE = cycloaddition-retroelectrocyclization; COSY = correlated spectroscopy; CV = cyclic voltammetry; DCM = dichloromethane; DCTB = *trans*-2-[3-(4-*tert*-butylphenyl)-2-methyl-2-propenylidene]malononitrile; DMA = *N*,*N*-dimethylaniline; DMF = dimethylformamide; EtOAc = ethyl acetate; Fc = ferrocene; HRLSI = high resolution laser spray ionization; HSQC = heteronuclear single quantum coherence; NOESY = nuclear Overhauser enhancement spectroscopy; MALDI-TOF = matrix-assisted laser desorption/ionization-time of flight; MS = mass spectrometry; NMR = nuclear magnetic resonance; ppm = part per million; PRC = pre-reactive complex; *n*-Bu_4NPF_6 = tetra-*n*-butylammonium hexafluorophosphate; TCNQ = tetracyanoquinodimethane; THF = tetrahydrofurane; TLC = thin layer chromatography.

1. Materials and methods

Chemicals and solvents were purchased from commercial suppliers (Aldrich, Fluka, Strem, Acros and Fischer) and used without further purification. All dry solvents were freshly distilled under argon over an appropriate drying agent before use. Column

chromatography was carried out on silica gel Merck-60 (230-400 mesh, 60 Å), and TLC on aluminum sheets precoated with silica gel 60 F254 (Merck).

MALDI-TOF MS spectra were obtained from a BRUKER ULTRAFLEX III instrument equipped with a nitrogen laser operating at 337 nm. NMR spectra were recorded with a BRUKER Avance-300, BRUKER AC-400 and a BRUKER DRX-500 instruments. The temperature was actively controlled at 298 K.

X-ray crystal diffraction analysis on anthryl-fused−*ext*TCNQ−DMA **1** and anthryl−*ext*TCNQ−DMA **5** was performed on a Bruker Kappa apparatus at 250 K (**1**) and 296 K (**5**) using two-dimensional detector Apex II with Mo kα radiation (λ = 0.71073 A). X-ray crystal diffraction analysis on anthryl−*ext*TCNQ−DMA **7** was performed on a XtaLAB Synergy R apparatus at 100 K using a HyPix-Arc 100 detector with Cu Kα radiation (λ = 1.54184 A). All the structures were solved by a direct method with the SHELXT program and refined by the SHELXL-2019/1 program. CCDC 2301908 (anthryl-fused−*ext*TCNQ−DMA **1**), CCDC 2301909 (anthryl−*ext*TCNQ−DMA **5**), and CCDC 2301910 (anthryl−*ext*TCNQ−DMA **7**).

UV-vis absorption spectra were recorded employing a JASCO-V660 spectrophotometer. The data were recorded with a scan rate of 400 nm/min.

NMR spectra were recorded with a Bruker AVANCE 300 (300 MHz), a Bruker AVANCE-II 300 (300 MHz), a BRUKER AVANCE III-HD Nanobay (300 MHz) and a Bruker DRX-500 (500 MHz) instruments in the Organic Chemistry Department of UAM and in the Interdepartmental Investigation Service of UAM.

Infrared spectra were recorded in the Interdepartmental Investigation Service of UAM on a Bruker IFS 66v vacuum FT-IR spectrometer.

Electrochemical measurements were performed in the Organic Chemistry Department of UAM on an Autolab PGStat 30 equipment using a three-electrode configuration system. The measurements were carried out in argon saturated THF solutions containing 0.1 M tetrabutylammonium hexafluorophosphate ($TBAPF_6^-$). A platinum electrode (3 mm diameter) was used as the working electrode, and a platinum wire and a $Ag/AgNO_3$ (0.01 M in acetonitrile) electrode were employed as the auxiliary and the reference electrode, respectively. Fc was used as an external reference and all the potentials were given relative to the Fc/Fc^+ couple.

Quantum mechanical calculations were performed at the Density Functional Theory level, using the Minnesota hybrid functional M06-2X,[1,2] in combination with the 6-31+G(d,p) basis set.

The M062X functional was shown to predict well thermochemistry data and a similar approach was fruitfully used in psat studies to investigate organic reaction mechanisms and cycloaddition mechanisms.[3,4] Intermediate states and transition states were confirmed by normal mode analysis. All chemical species were optimised separately in both gas phase and condensed phase, using implicit solvation models. All calculations were performed using the Gaussian16 software package.[5] Binding energies (ΔE) were computed using the super-molecular approach (i.e., for the reaction: A + B → AB, $\Delta E_{AB} = E_{AB} - E_A - E_B$) with the inclusion of the zero-point energy. Moreover, the effect of the solvent on the adduct binding energy was evaluated using the polarizable continuum model (PCM)[6] considering THF as a solvent, as implemented in Gaussian16. Reaction enthalpies and free energies of the addition reaction were estimated from binding energy calculations including thermal effects and the solvent contribution.

The reactivity of molecular sites of different derivatives was analysed using condensed Fukui function (CFF),[7] since it was proved to be a successful tool in studying reactive sites.[8] CFF describes the electron density in a frontier orbital thus, a change in the total number of electrons. For a given atom, i, in a molecule with N electrons in a constant external potential, V(r), the CFF for nucleophilic (*i.e.*, f_i^-) and electrophilic (*i.e.*, f_i^+) attack can be obtained from finite difference approximation (i.e., ± 1e) as:

$$f_i^+ = [q_i(N) - q_i(N+1)] \quad (1)$$

$$f_i^- = [q_i(N-1) - q_i(N)] \quad (2)$$

where $q_i(N)$, $q_i(N+1)$, $q_i(N-1)$ are the atomic charges for the i-th atom in the neutral, reduced and oxidized states of the molecule, respectively.

The Hirshfeld population analysis[9] was used to evaluate the atomic charges since it has been suggested to give the optimal atomic charges to perform CFF analysis.[10]

9-(4-*N*,*N*-dimethylaminophenyl)-ethynylanthracene **3** (Figure S1.1) was prepared according to a previously reported synthetic procedure showing identical spectroscopic properties to that reported therein.[11]

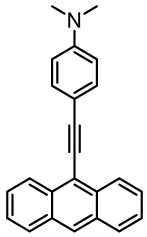

3

Figure S1.1. Molecular structure of 9-(4-*N*,*N*-dimethylaminophenyl)-ethynylanthracene **3**.

9-Bromo-10-mesitylanthracene (Figure S1.2) was prepared according to a previously reported synthetic procedure showing identical spectroscopic properties to that reported therein.[12]

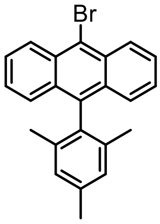

Figure S1.2. Molecular structure of 9-bromo-10-mesitylanthracene.

2. Mechanistic insight on the [2+2] cycloaddition-retroelectrocyclization (CA-RE) reaction between a generic, activated unsymmetric alkyne and TCNQ

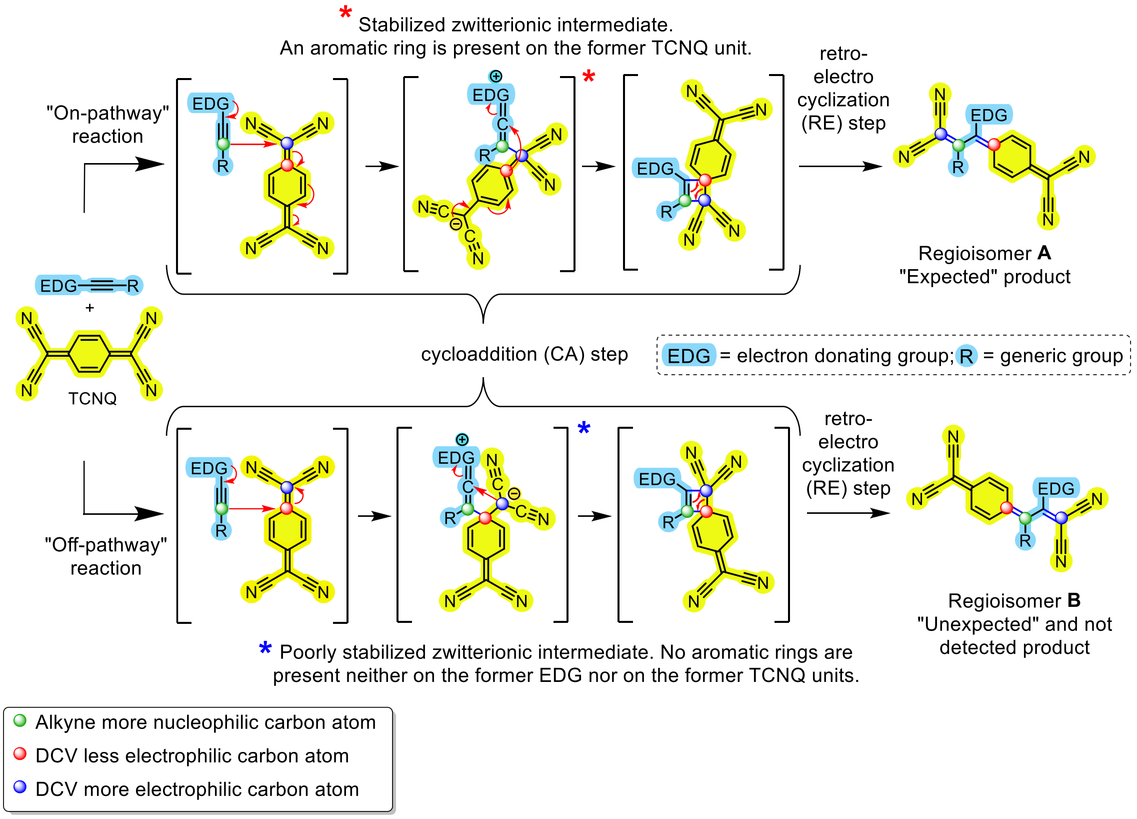

Scheme S2.1. "On-pathway" (top) and "off-pathway" (bottom) reaction for the stepwise, non-concerted [2+2] cycloaddition-retro-electrocyclization (CA-RE) reaction between a generic activated unsymmetric alkyne (colored in blue) and TCNQ (colored in yellow) leading to the "expected" and "unexpected" regioisomers **A** and **B**, respectively. In the two regioisomers as well as in the reaction intermediate species, the molecular fragments derived from the alkyne and the TCNQ moieties have been colored in blue and yellow, respectively. Newly formed bonds are colored in blue. Red arrows show electrons' movement. For an easier identification of the structural rearrangement occurring throughout the CA-RE reaction, the two carbon atoms of the exocyclic double bond of TCNQ and the more nucleophilic carbon atom of the alkyne moiety have been marked with a red (*i.e.*, the less electrophilic carbon atom of the DCV unit), blue (*i.e.*, the more electrophilic carbon atom of the DCV unit), and green dot, respectively. Blue and red asterisks refer to zwitterionic intermediates with no aromatic and aromatic ring at the former TCNQ six-member ring, respectively.

3. Synthesis and characterization of anthryl-fused−*ex*TCNQ−DMA 1 and anthryl−*ex*TCNQ−DMA derivatives 5 and 7

Synthesis and characterization of anthryl−extTCNQ−DMA 2

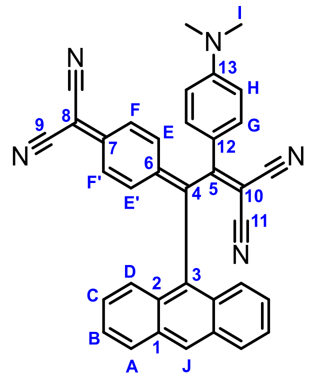

9-(4-*N*,*N*-dimethylaminophenyl)-ethynylanthracene **3** (21.2 mg, 0.066 mmol) was loaded in a 25 mL round-bottomed flask and dissolved in THF (2 mL). TCNQ (40.4 mg, 0.198 mmol) was added to that solution and the mixture was stirred at r.t. over 16 h. The solvent was removed under reduced pressure and the mixture was subjected to column chromatography (SiO_2 gel, eluent = DCM), where an intense blue band was collected. The resulting crude product was suspended in *n*-hexane, sonicated, filtered and washed with some more *n*-hexane, collected and dried under vacuum to yield anthryl−*ext*TCNQ−DMA **2** (26 mg, 47%)* as a dark blue solid.

^{1}H-NMR (400 MHz, $CDCl_3$): δ 8.60 (s, 1H, H_J), 8.05 (d, J = 7.5 Hz, 2H, H_A), 7.83 (d, J = 9.3 Hz, 2H, H_D), 7.58 – 7.48 (m, 4H, H_{B+C}), 7.40 (d, J = 9.1 Hz, 2H, H_G), 7.37 (dd, J = 9.8, 1.9 Hz, 1H, H_E), 7.29 (dd, J = 9.8, 1.9 Hz, 1H, H_F), 6.98 (dd, J = 9.7, 2.0 Hz, 1H, $H_{F'}$), 6.61 (dd, J = 9.7, 1.9 Hz, 1H, $H_{E'}$), 6.46 (d, J = 9.1 Hz, 2H, H_H), 2.99 (s, 6H, H_I); **^{13}C-NMR** (126 MHz, $CDCl_3$) δ (ppm) = 170.4 (C_5), 165.6, 165.5, 153.9 ($C_{6/7}$), 153.4 (C_{13}), 149.2 (C_4), 143.8 ($C_{6/7}$), 137.0 ($C_{E'}$), 134.3 (C_E), 134.2, 133.9, 133.8, 132.6 (C_J), 132.4 (C_G), 131.3, 131.1, 130.4, 129.9, 129.7 (C_A), 129.0, 128.4 (C_B), 126.9 (C_F), 126.2 ($C_{F'}$), 125.9 (C_C), 125.0 (C_D), 123.4 (C_{12}), 111.5 (C_H), 81.8 (C_{10}), 79.5 (C_8), 40.1 (C_I); **MALDI-TOF** (DCTB, negative mode): *m/z* 525.1941−527.1970 $[M]^{\bullet-}$; **HRLSI-MS**: Calculated for $C_{36}H_{23}N_5$: 525.1959; Found: 525.1941; **UV/vis** ($CHCl_3$): λ_{max} (nm) (log ε)

* It is important to notice that the moderate yield is due to the fact that the CA-RE reaction is carried out at r.t. in order minimize the transformation of anthryl−*ext*TCNQ−aniline **2** into its fused analogue anthryl-fused−*ext*TCNQ−aniline **1**.

= 676 (4.24), 406 (4.83). **FT-IR** (ATR) ν (cm^{-1}) = 2212 (C≡N), 2117, 1596, 1488, 1426, 1367, 1310, 1185, 1105, 1017, 945, 895, 806, 730, 590.

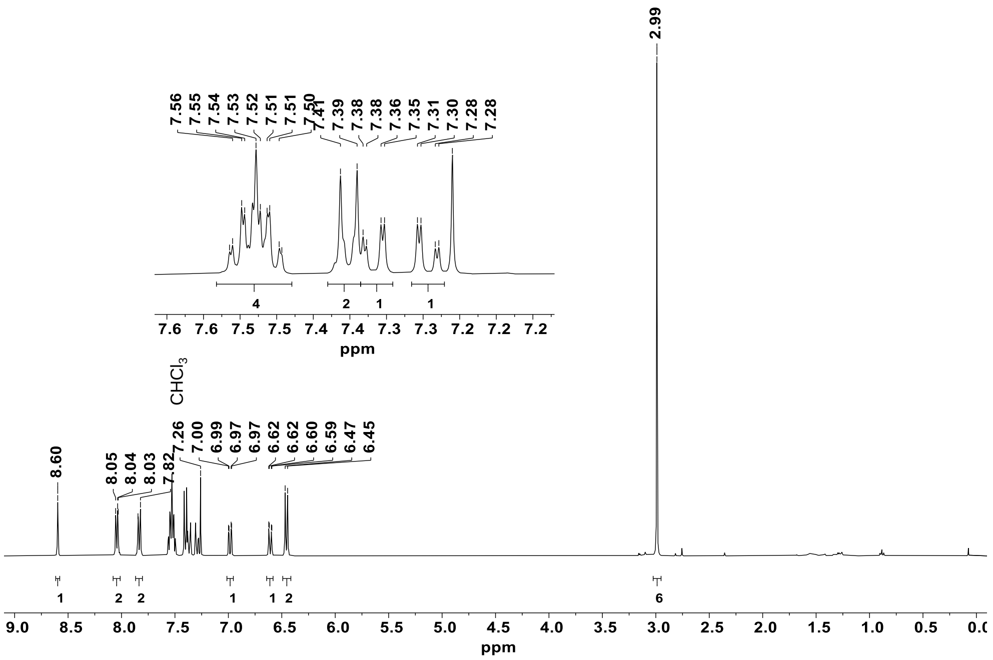

Figure S3.1. ^{1}H-NMR spectrum of anthryl−*ext*TCNQ−DMA **2** in $CDCl_3$.

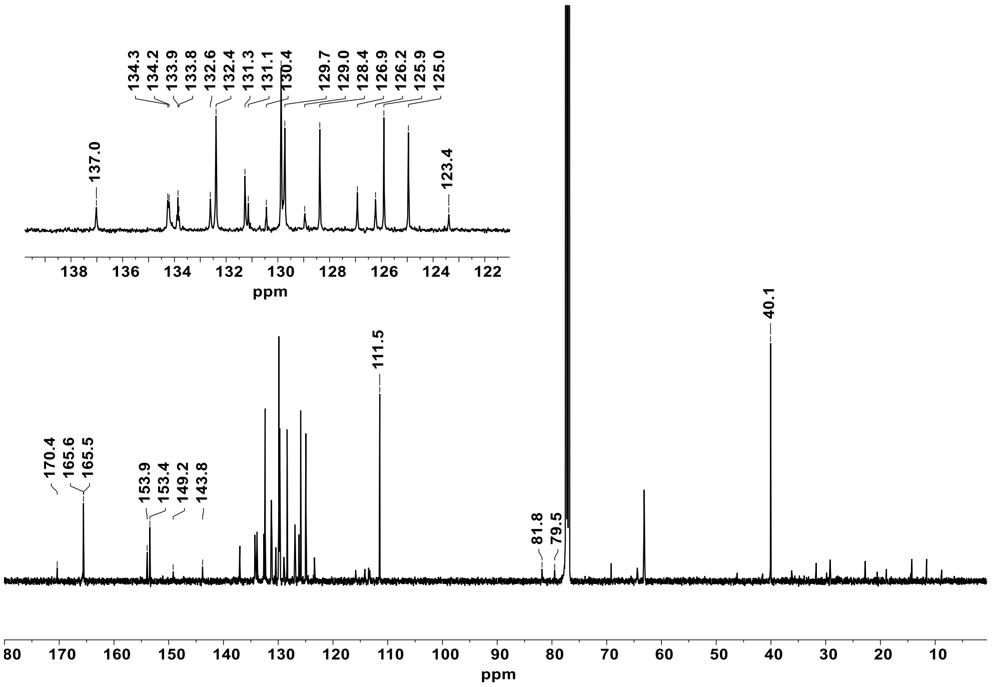

Figure S3.2. ^{13}C-NMR spectrum of anthryl−*ext*TCNQ−DMA **2** in $CDCl_3$.

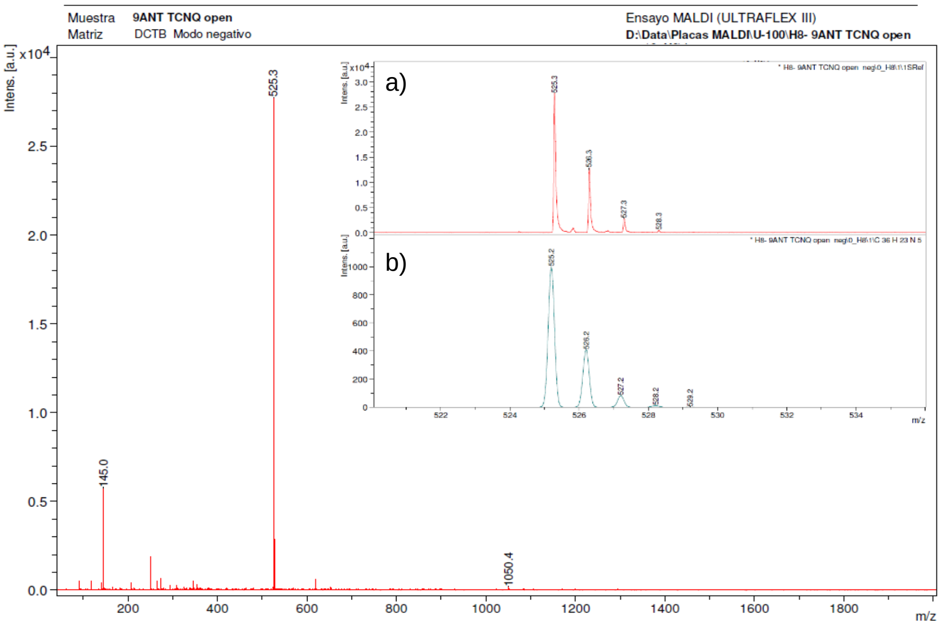

Figure S3.3. MALDI-TOF mass spectrum (DCTB matrix) of anthryl−*ext*TCNQ−DMA **2**. Inset: a) Isotopic distribution of the MALDI peaks between 521 and 535 m/z; b) calculated isotopic pattern for anthryl−*ext*TCNQ−DMA **2**.

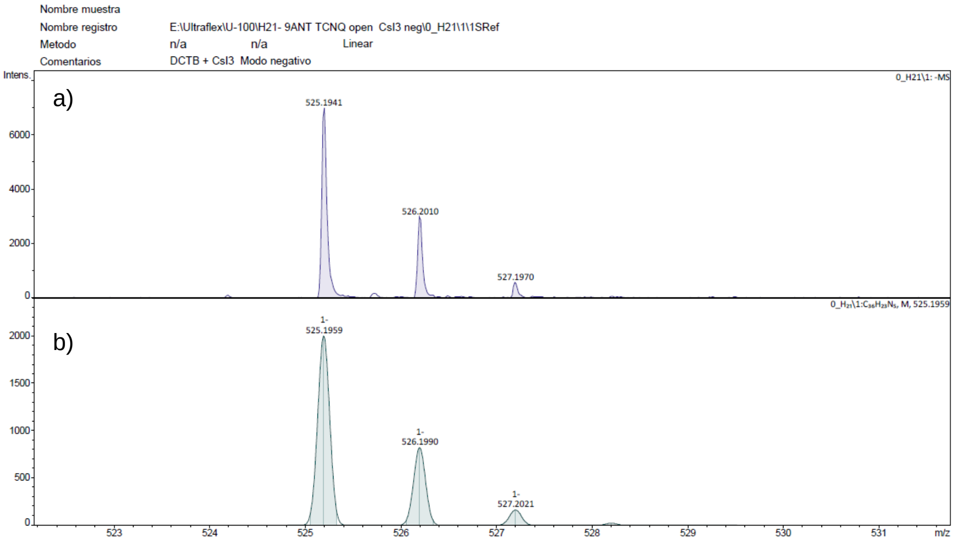

Figure S3.4. a) HRLSI-MS spectrum of anthryl−*ext*TCNQ−DMA **2** between 522 and 532 m/z; b) calculated isotopic pattern for anthryl−*ext*TCNQ−DMA **2**.

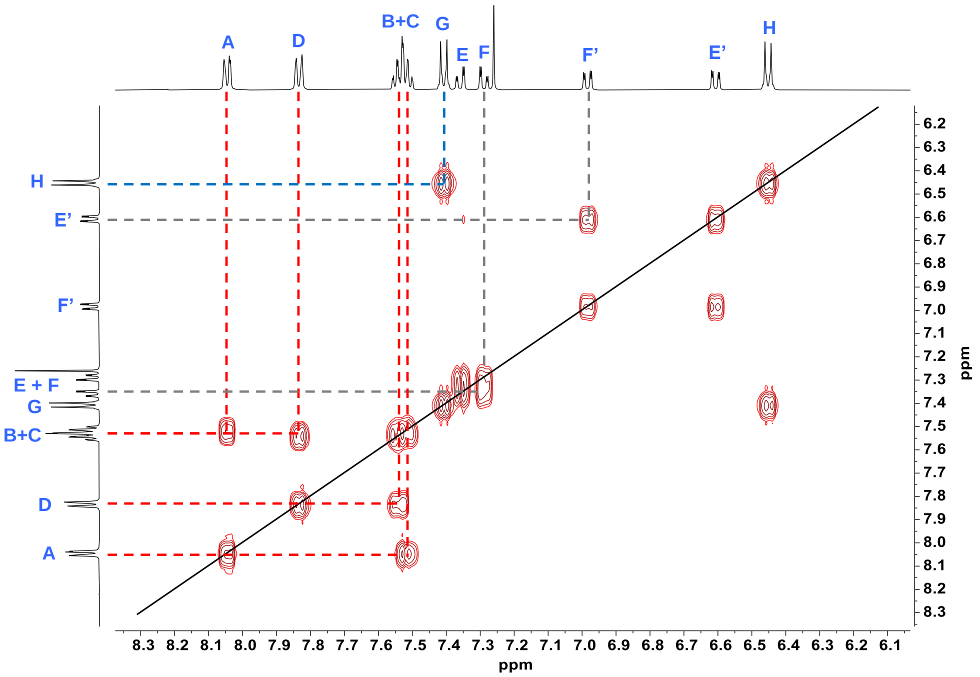

Figure S3.5. ^{1}H-COSY-NMR spectrum of anthryl−*ext*TCNQ−DMA **2** in $CDCl_3$ highlighting the correlations between the anthryl protons (red dashed lines), the protons of the DMA moiety (blue dashed lines) and the protons of the (dicyanomethylene)cyclohexa-2,5-diene fragment (grey dashed lines).

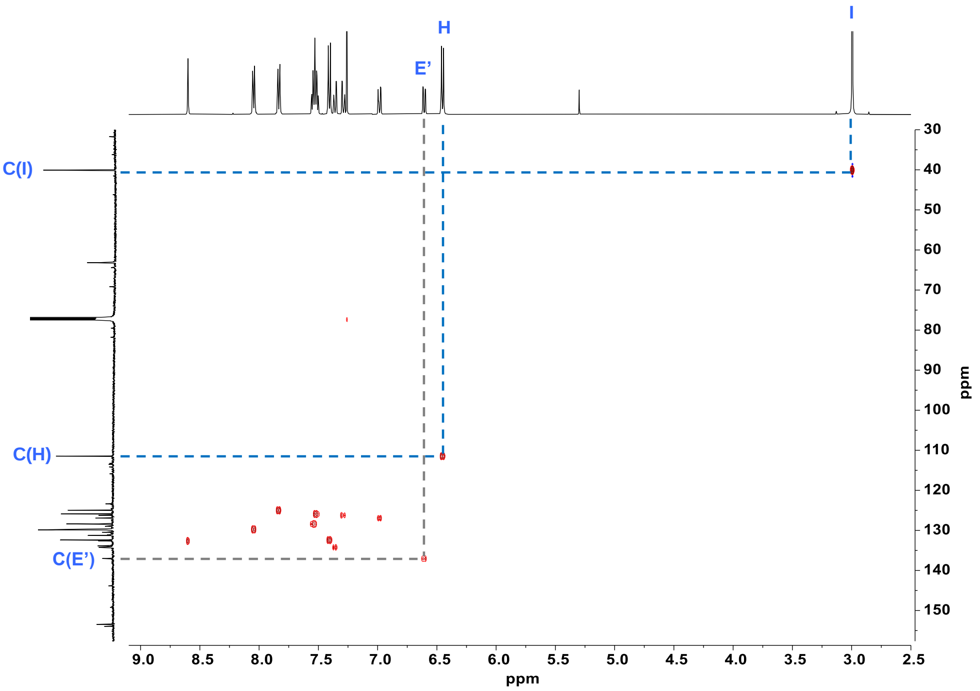

Figure S3.6. $^{13}C\{^{1}H\}$ HSQC-NMR spectrum of anthryl−*ext*TCNQ−DMA **2** in $CDCl_3$ highlighting the correlations between the anthryl protons (red dashed lines), the protons of the

DMA moiety (blue dashed lines) and the protons of the (dicyanomethylene)cyclohexa-2,5-diene fragment (grey dashed lines).

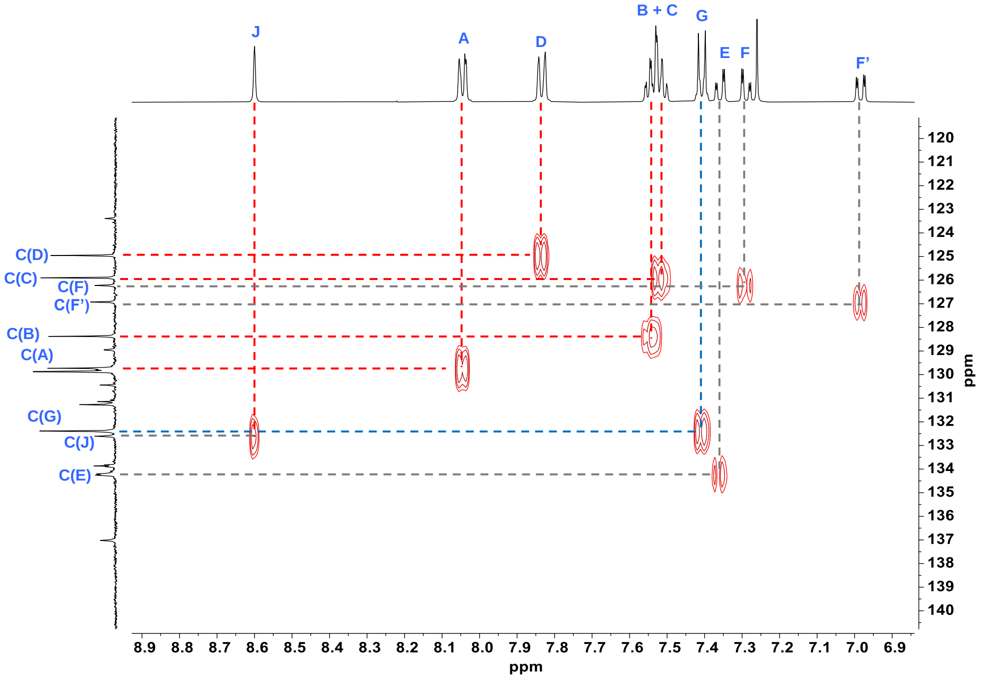

Figure S3.7. Portion of the $^{13}C\{^{1}H\}$ HSQC-NMR spectrum of anthryl−*ext*TCNQ−DMA **2** in $CDCl_3$ highlighting the correlations between the anthryl protons (red dashed lines), the protons of the DMA moiety (blue dashed lines) and the protons of the (dicyanomethylene)cyclohexa-2,5-diene fragment (grey dashed lines).

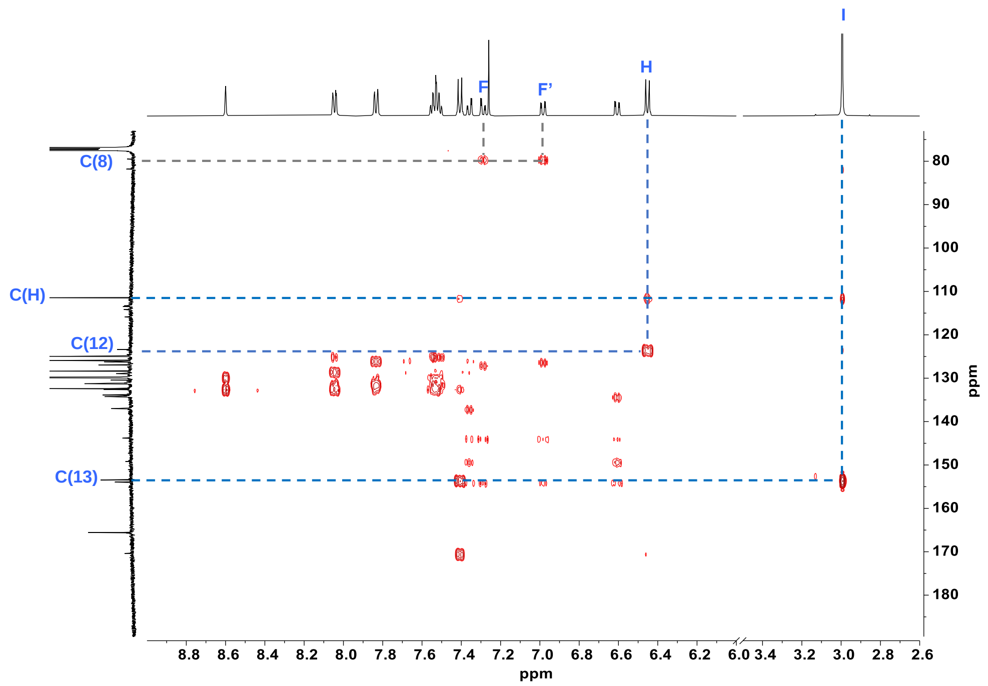

Figure S3.8. $^{13}C\{^{1}H\}$ HMBC-NMR spectrum of anthryl–*ext*TCNQ–DMA **2** in $CDCl_3$ highlighting the correlations between the anthryl protons (red dashed lines), the protons of the DMA moiety (blue dashed lines) and the protons of the (dicyanomethylene)cyclohexa-2,5-diene fragment (grey dashed lines).

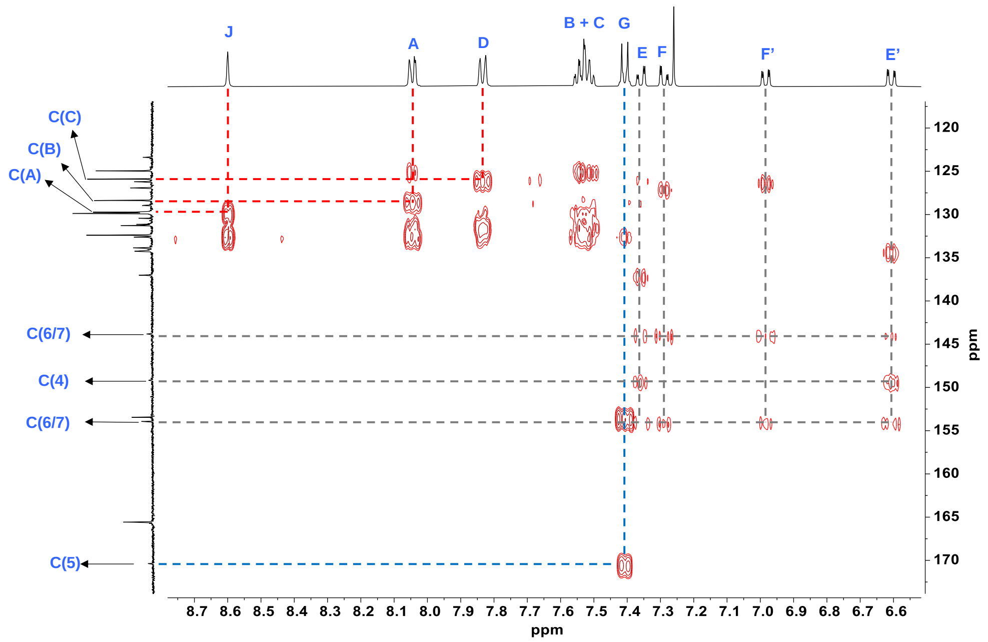

Figure S3.9. Portion of the $^{13}C\{^{1}H\}$ HMBC-NMR spectrum of anthryl–*ext*TCNQ–DMA **2** in $CDCl_3$ highlighting the correlations between the anthryl protons (red dashed lines), the protons of

the DMA moiety (blue dashed lines) and the protons of the (dicyanomethylene)cyclohexa-2,5-diene fragment (grey dashed lines).

Synthesis and characterization of anthryl-fused−extTCNQ−DMA 1

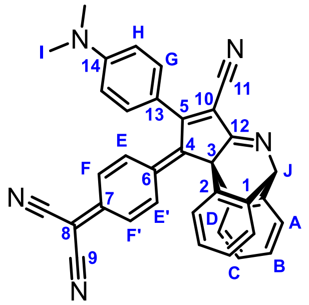

Anthryl−*ext*TCNQ−DMA **2** (95 mg, 0.1807 mmol) was loaded in a 10 mL round-bottom flask, dissolved in toluene (8 mL) and degassed with argon for several minutes. Then, the reaction was heated to 90 °C and stirred over 16 h. The solvent was removed under reduced pressure and the mixture was subjected to column chromatography (SiO_2 gel, eluent = DCM to DCM:EtOAc 10:1), where the second intense red band was collected. The solvent was removed under reduced pressure and the crude product was subjected to size exclusion chromatography (BioBeads, $CHCl_3$) and the solvents were then removed under reduced pressure. The resulting crude product was suspended in *n*-hexane, sonicated, filtered and washed with some more *n*-hexane, collected and dried under vacuum to yield anthryl-fused−*ext*TCNQ−DMA **1** (88.3 mg, 93%) as a dark red solid.

^{1}H-NMR (400 MHz, $CDCl_3$): δ 7.59 (d, *J* = 8.7 Hz, 2H, H_A), 7.50 (dd, *J* = 9.9, 2.0 Hz, 1H, $H_{E/E'}$), 7.40 (d, *J* = 8.9 Hz, 2H, H_G), 7.31 (dd, *J* = 9.8, 2.0 Hz, 1H, $H_{E/E'}$), 7.24 (dd, *J* = 7.5, 1.2 Hz, 2H, H_B), 7.21 (dd, *J* = 9.8, 2.1 Hz, 1H, $H_{F/F'}$), 7.17 (dd, *J* = 9.9, 2.0 Hz, 1H, $H_{F/F'}$), 7.13 (td, *J* = 7.5, 1.2 Hz, 2H, H_C), 6.99 (d, *J* = 7.5 Hz, 2H, H_D), 6.86 (s, 1H, H_J), 6.82 (d, *J* = 9.0 Hz, 2H, H_H), 3.13 (s, 6H, H_I); **^{13}C-NMR** (75 MHz, $CDCl_3$) δ (ppm) = 178.9 (C_{12}), 168.2 (C_5), 153.5 (C_7), 152.4 (C_{14}), 147.6 (C_6), 143.6 (C_2), 137.9 ($C_{E/E'}$), 137.3 (C_1), 133.8, 133.4 ($C_{E/E'}$), 130.0 (C_G), 127.4 (C_B), 126.6 (C_C), 125.9 ($C_{F/F'}$), 125.7 ($C_{F/F'}$), 124.5 (C_A), 123.6 (C_D), 119.5 (C_{13}), 116.7, 113.6, 113.5, 113.4, 112.1 (C_H), 79.6 (C_8), 72.3 (C_J), 64.4 (C_3), 40.2 (C_I); **MALDI-TOF** (DCTB, negative mode): *m/z* 525.1965−527.2071 $[M]^{\bullet-}$; **HRLSI-MS**: Calculated for $C_{36}H_{23}N_5$: 525.1959; Found: 525.1965; **UV/vis** ($CHCl_3$): λ_{max} (nm) (log ε) = 700 (broad), 538 (sh), 499 (4.33), 469

(sh), 377 (4.11); **FT-IR** (ATR) ν (cm^{-1}) = 2976, 2210 (C≡N), 1736, 1605, 1541, 1495, 1442, 1364, 1197, 826, 746.

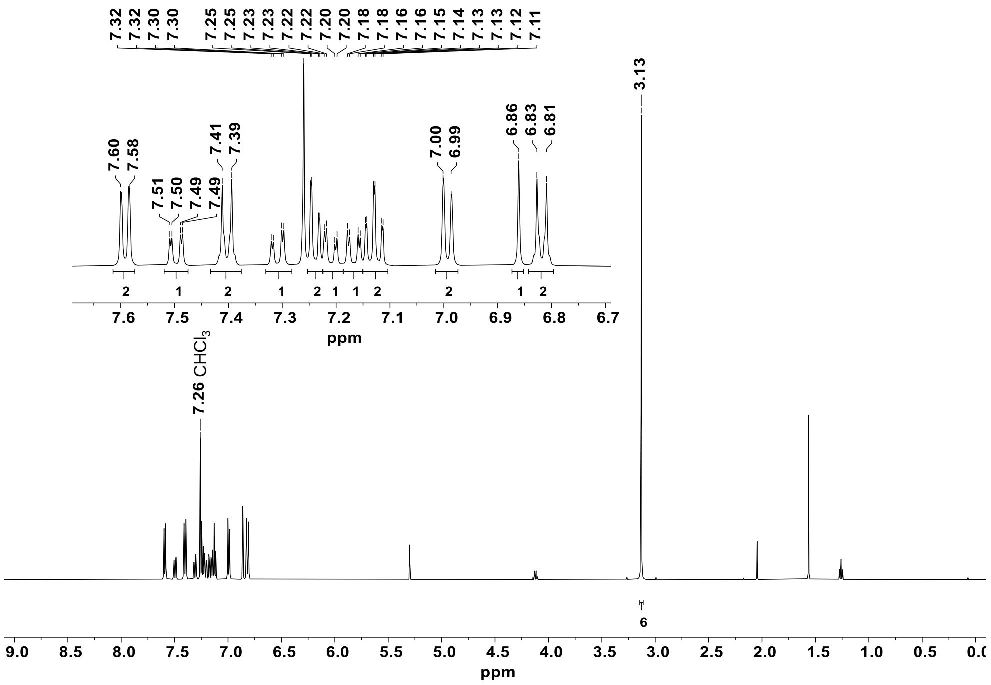

Figure S3.10. ^{1}H-NMR spectrum of anthryl-fused−*ext*TCNQ−DMA **1** in $CDCl_3$.

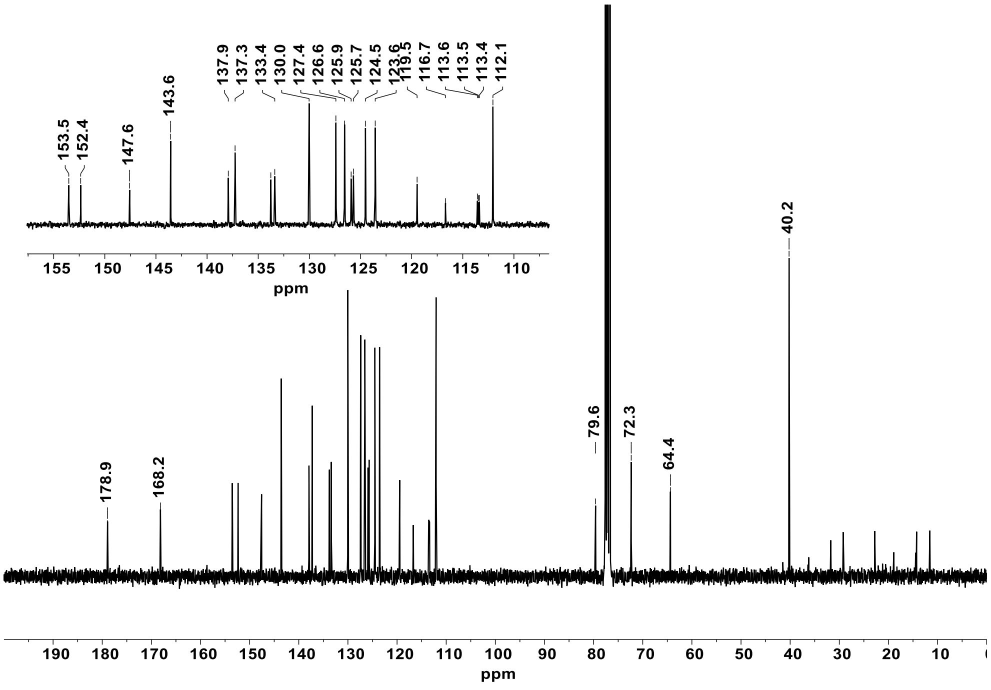

Figure S3.11. ^{13}C-NMR spectrum of anthryl-fused−*ext*TCNQ−DMA **1** in $CDCl_3$.

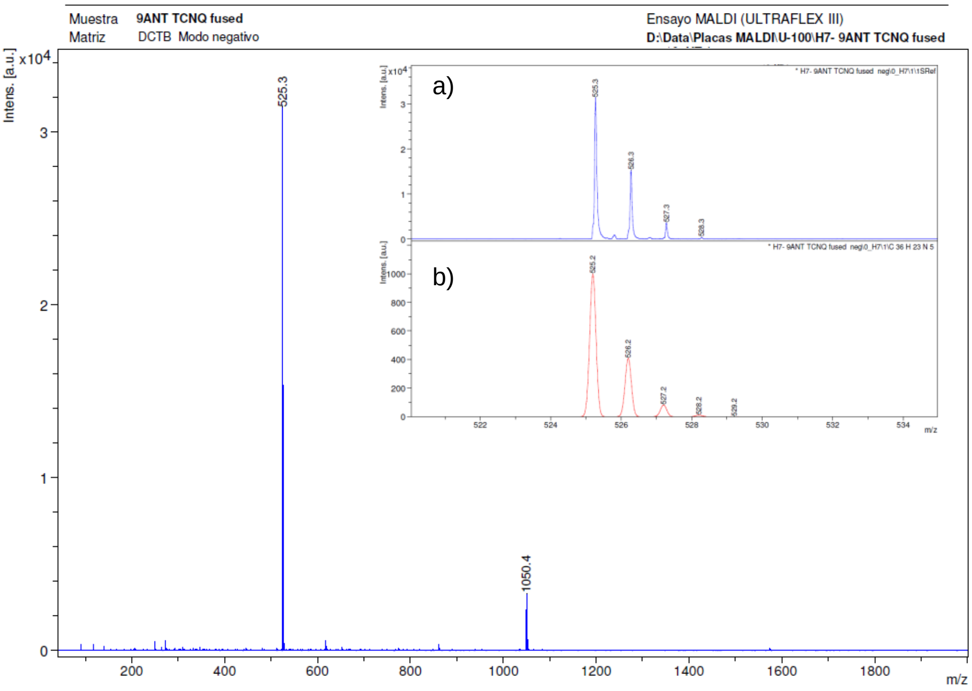

Figure S3.12. MALDI-TOF mass spectrum (DCTB matrix) of anthryl-fused−*ext*TCNQ−DMA **1**. Inset: a) Isotopic distribution of the MALDI-TOF peaks between 521 and 534 m/z; b) calculated isotopic pattern for anthryl-fused−*ext*TCNQ−DMA **1**.

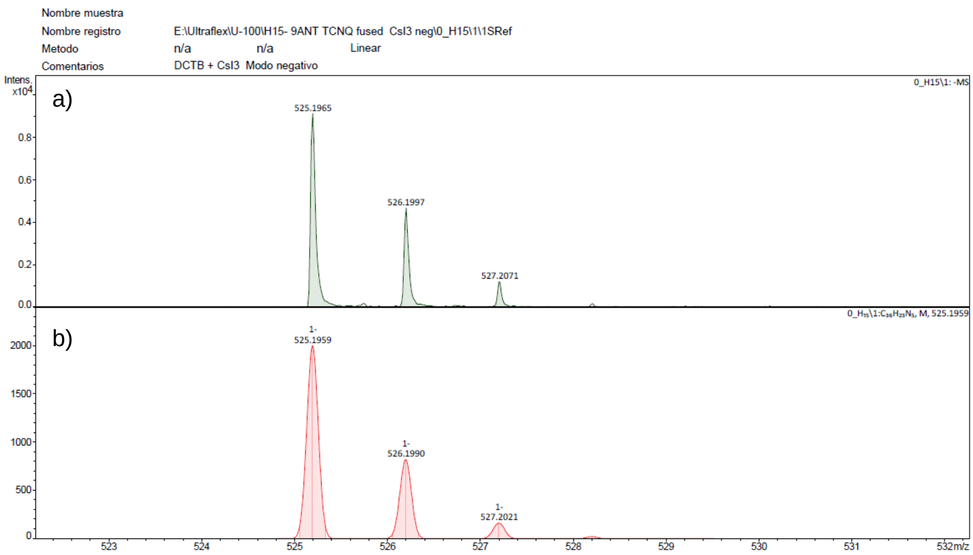

Figure S3.13. a) HRLSI-MS spectrum of anthryl-fused−*ext*TCNQ−DMA **1**; b) calculated isotopic pattern for anthryl-fused−*ext*TCNQ−DMA **1**.

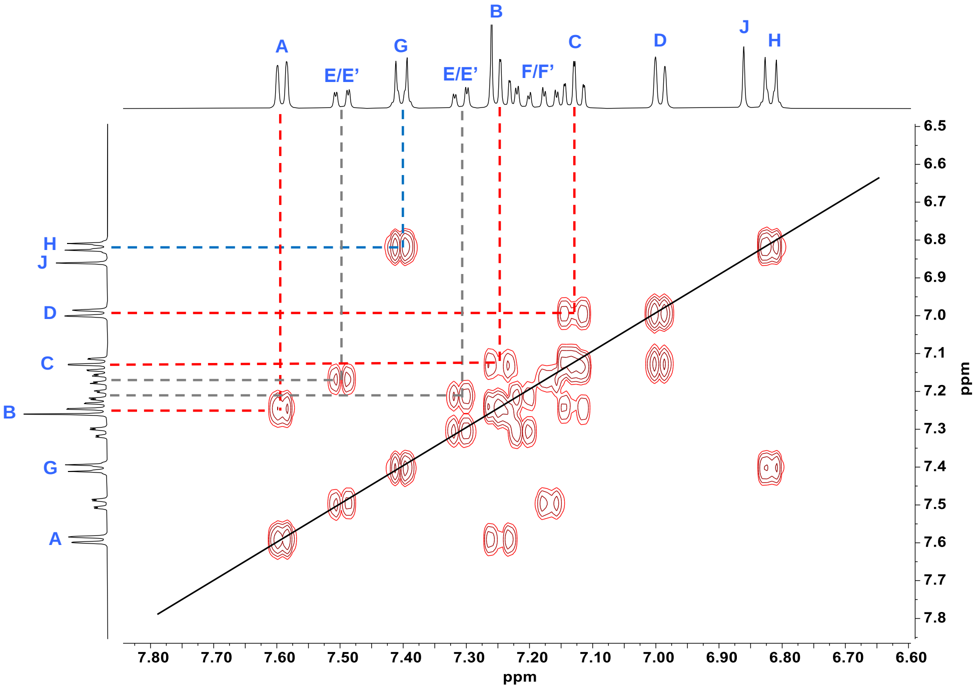

Figure S3.14. ^{1}H-COSY-NMR spectrum of anthryl-fused−*ext*TCNQ−DMA **1** in $CDCl_3$ highlighting the correlations between the anthryl protons (red dashed lines), the protons of the DMA moiety (blue dashed lines) and the protons of the (dicyanomethylene)cyclohexa-2,5-diene fragment (grey dashed lines).

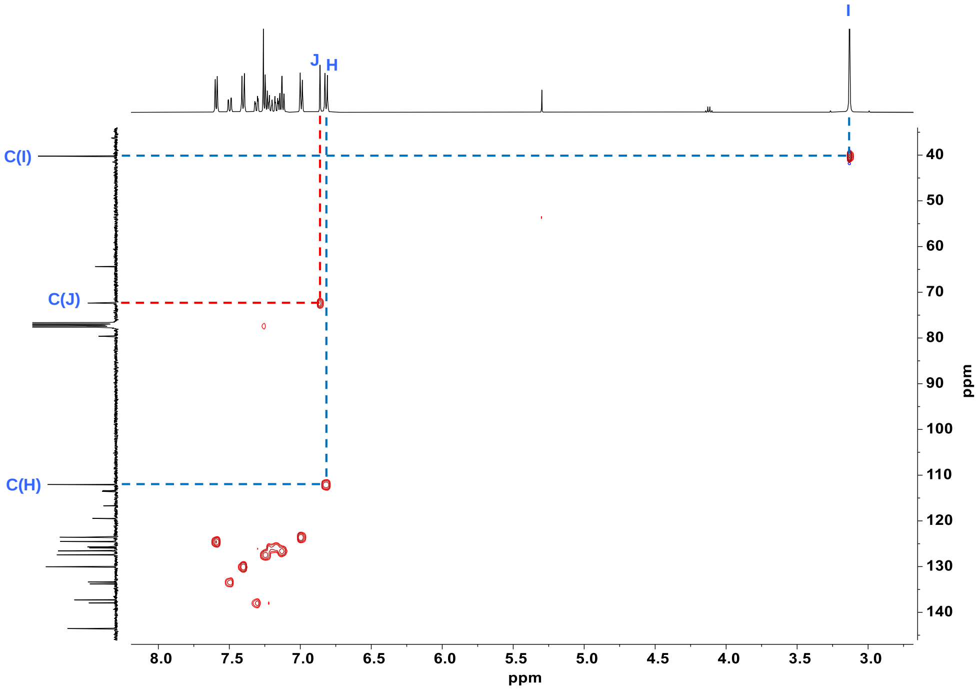

Figure S3.15. ^{13}C{^{1}H} HSQC-NMR spectrum of anthryl-fused−*ext*TCNQ−DMA **1** in $CDCl_3$ highlighting the correlations between the anthryl protons (red dashed lines), the protons of the

DMA moiety (blue dashed lines) and the protons of the (dicyanomethylene)cyclohexa-2,5-diene fragment (grey dashed lines).

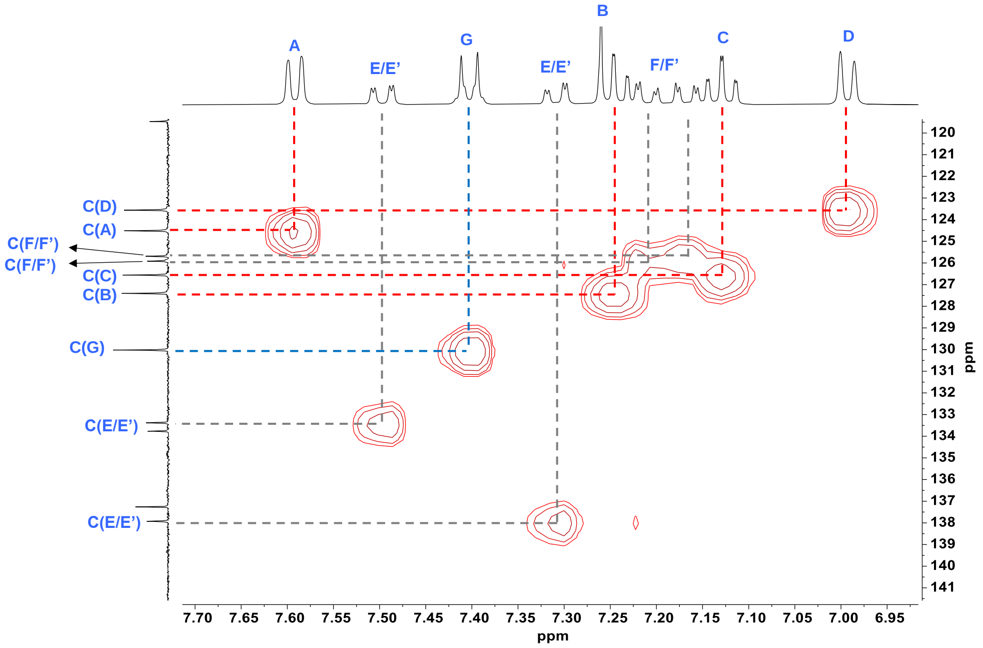

Figure S3.16. Portion of the $^{13}C\{^1H\}$ HSQC-NMR spectrum of anthryl-fused−*ext*TCNQ−DMA **1** in $CDCl_3$ highlighting the correlations between the anthryl protons (red dashed lines), the protons of the DMA moiety (blue dashed lines) and the protons of the (dicyanomethylene)cyclohexa-2,5-diene fragment (grey dashed lines).

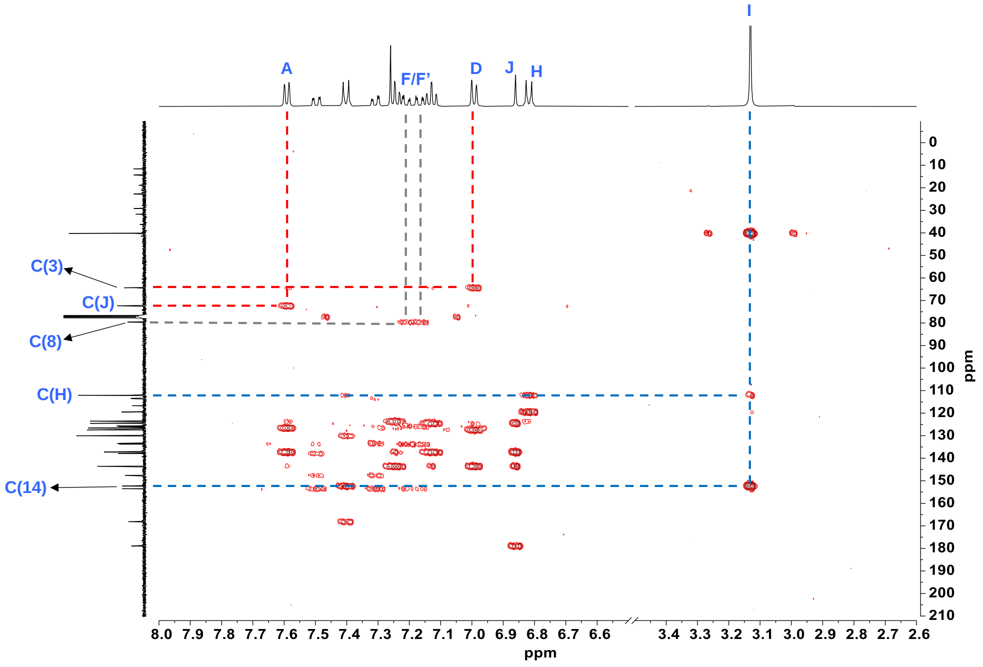

Figure S3.17. $^{13}C\{^{1}H\}$ HMBC-NMR spectrum of anthryl-fused−*ext*TCNQ−DMA **1** in $CDCl_3$ highlighting the correlations between the anthryl protons (red dashed lines), the protons of the DMA moiety (blue dashed lines) and the protons of the (dicyanomethylene)cyclohexa-2,5-diene fragment (grey dashed lines).

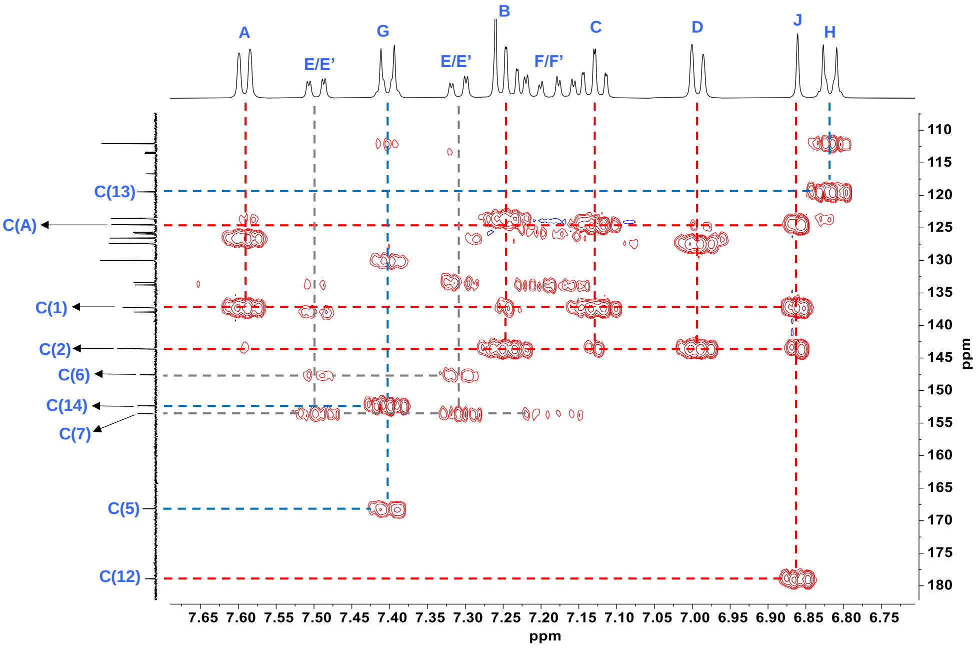

Figure S3.18. Portion of the $^{13}C\{^{1}H\}$ HMBC-NMR spectrum of anthryl-fused−*ext*TCNQ−DMA **1** in $CDCl_3$ highlighting the correlations between the anthryl protons (red dashed lines), the protons of the DMA moiety (blue dashed lines) and the protons of the (dicyanomethylene)cyclohexa-2,5-diene fragment (grey dashed lines).

Table S3.1. Selected crystallographic data for anthryl-fused−*ext*TCNQ−DMA **1**.

**Chemical formula**	$C_{36}H_{23}N_5$
**Formula weight**	525.59 g/mol
**Temperature**	250(2) K
**Wavelength**	0.71073 Å
**Crystal size**	0.133 × 0.273 × 0.414 mm
**Crystal habit**	clear intense blue plate
**Crystal system**	monoclinic
**Space group**	P 1 21/c 1

**Unit cell dimensions**	a = 14.7514(4) Å	α = 90°
	b = 11.4817(3) Å	β = 101.8410(10)°
	c = 16.3505(5) Å	γ = 90°
**Volume**	2710.37(13) $Å^3$	
**Z**	4	
**Density (calculated)**	1.288 g/cm^3	
**Absorption coefficient**	0.078 mm^{-1}	
**F(000)**	1096	
**Theta range for data collection**	3.55 to 25.35°	
**Index ranges**	–17<=h<=17, –13<=k<=13, –19<=l<=19	
**Reflections collected**	132068	
**Independent reflections**	4946 [R(int) = 0.0418]	
**Coverage of independent reflections**	99.7%	
**Absorption correction**	Multi-Scan	
**Max. and min. transmission**	0.9900 and 0.9690	
**Structure solution technique**	direct methods	
**Structure solution program**	XT, VERSION 2018/2	
**Refinement method**	Full-matrix least-squares on F^2	
**Refinement program**	SHELXL-2019/1 (Sheldrick, 2019)	
**Function minimized**	$\Sigma w(F_o^2 - F_c^2)^2$	
**Data / restraints / parameters**	4946 / 0 / 372	
**Goodness-of-fit on F^2**	1.057	
**Final R indices**	4201 data; I>2σ(I)	R1 = 0.0380, wR2 = 0.1028
	all data	R1 = 0.0463, wR2 = 0.1102

**Weighting scheme**	$w=1/[\sigma^2(F_o^2)+(0.0571P)^2+0.7351P]$ where $P=(F_o^2+2F_c^2)/3$
**Largest diff. peak and hole**	0.217 and -0.193 eÅ^{-3}
**R.M.S. deviation from mean**	0.044 eÅ^{-3}

Synthesis and characterization of 1-(4-N,N-dimethylaminophenyl)-ethynylanthracene 4

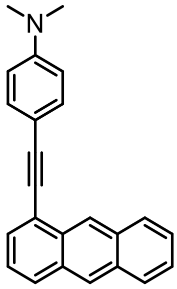

1-Bromoanthracene (30 mg, 0.117 mmol) and $Pd(PPh_3)_4$ (6.8 mg, 0.00585 mmol, 5 mol%) were added subsequently to a dried 50 mL Schlenk tube and subjected to three cycles vacuum and argon backfilling each. Then, under argon, dry DMF (3 mL) and NEt_3 (1.5 mL) were added, and the mixture was stirred. Then, sodium ascorbate (1.39 mg, 0.00702 mmol, 6 mol%) was added to the reaction mixture and it was stirred until dissolved. After this $CuSO_4\cdot5H_2O$ (0.58 mg, 0.00234 mmol, 2 mol%) was added. Finally, 4-ethynyl-*N*,*N*-dimethylaniline (22.1 mg, 0.1521 mmol) was dissolved in dried DMF (0.5 mL) under argon and the solution added to the reaction mixture dropwise. Then, the mixture was stirred at 80 ºC for 16 h. The reaction mixture was then allowed to reach r.t. and the crude was dissolved in EtOAc (25 mL) and washed with a saturated solution of NH_4Cl (25 mL), H_2O (2 × 15 mL), and brine (15 mL). After this, the organic layer was dried with anhydrous Na_2SO_4. The solution was filtered, and the solvent was removed under reduced pressure. The solid was subjected to column chromatography (SiO_2 gel, 1:1 $CHCl_3$/*n*-heptane) and an intense yellow-coloured, fluorescent band was collected. The solvents were evaporated under reduced pressure. The resulting crude product was suspended in MeOH, sonicated, filtered and washed with some more MeOH, collected and dried under vacuum to yield 1-(4-*N*,*N*-dimethylaminophenyl)-ethynylanthracene **4** (27.3 mg, 73%) as a yellow solid.

^{1}H-NMR (300 MHz, $CDCl_3$): δ 9.02 (s, 1H), 8.44 (s, 1H), 8.16 – 8.07 (m, 1H), 8.06 – 7.98 (m, 1H), 7.96 (d, *J* = 8.7 Hz, 1H), 7.73 (d, J = 6.8 Hz, 1H), 7.60 (d, J = 9.0 Hz, 2H), 7.54 – 7.46 (m, 2H), 7.47 – 7.38 (m, 1H), 6.75 (d, J = 8.7 Hz, 2H), 3.04 (s, 6H); **^{13}C-NMR** (126 MHz, $CDCl_3$) δ (ppm) = 150.4, 133.8, 133.0, 132.1, 132.0, 131.5, 131.3, 129.5, 128.8, 128.4, 128.1, 126.9, 125.8, 125.7, 125.4, 125.0, 122.0, 112.1, 111.9, 110.4, 96.3, 85.9, 40.4; **MALDI-TOF** (DCTB, positive mode): *m/z* 321.1500–323.1571 $[M]^{\bullet+}$; **HRLSI-MS**: Calculated for $C_{24}H_{19}N_1$: 321.1512; Found: 321.1500; **UV/vis** ($CHCl_3$): λ_{max} (nm) (log ε) = 413 (sh), 397 (4.40).

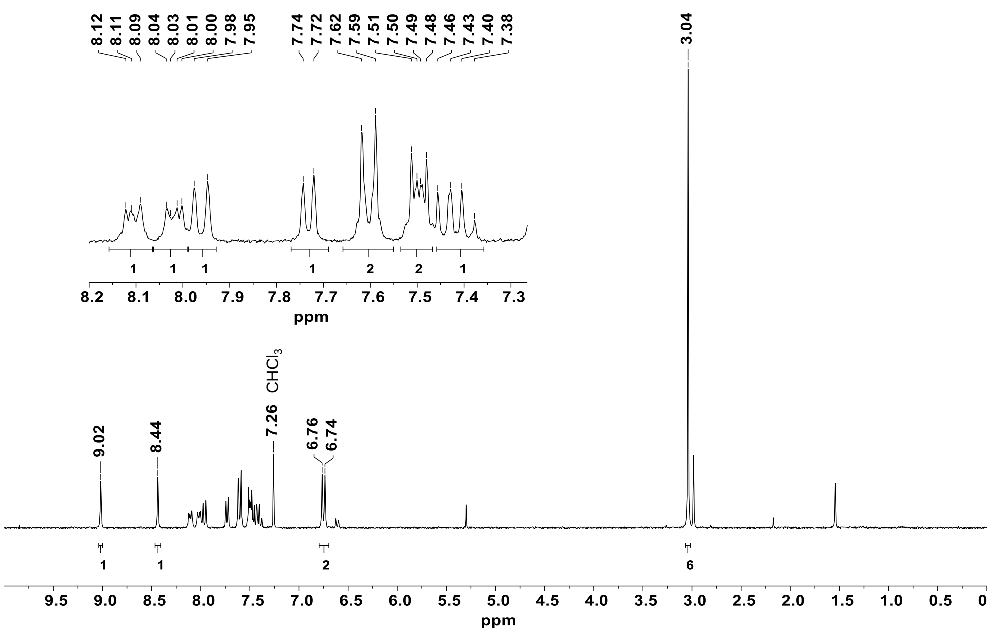

Figure S3.19. ^{1}H-NMR spectrum of 1-(4-*N*,*N*-dimethylaminophenyl)-ethynylanthracene **4** in $CDCl_3$.

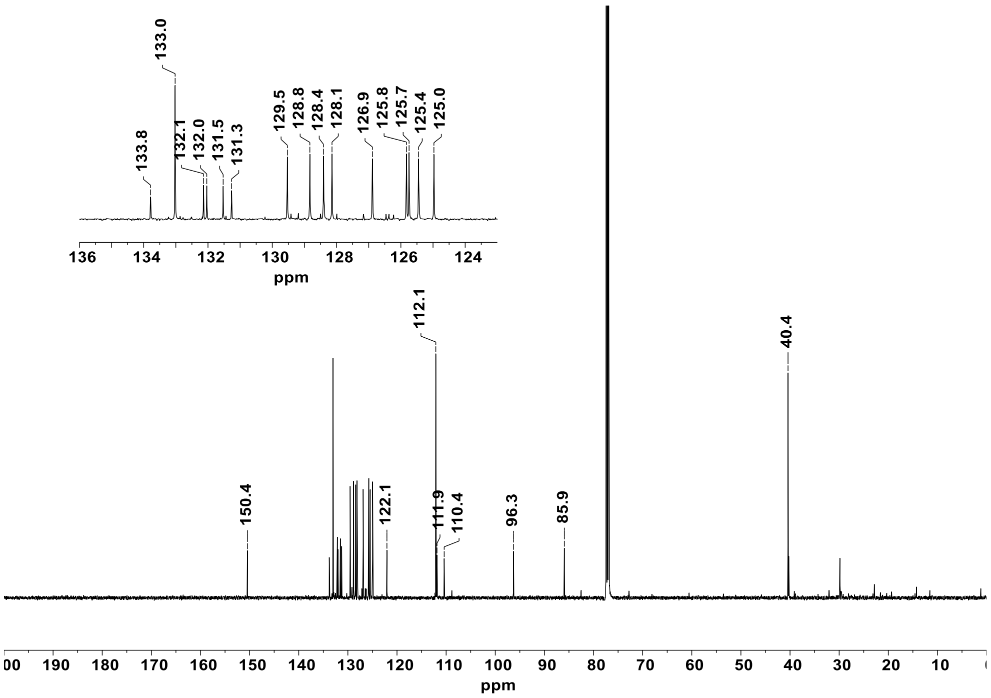

Figure S3.20. ^{13}C-NMR spectrum of 1-(4-*N*,*N*-dimethylaminophenyl)-ethynylanthracene **4** in $CDCl_3$.

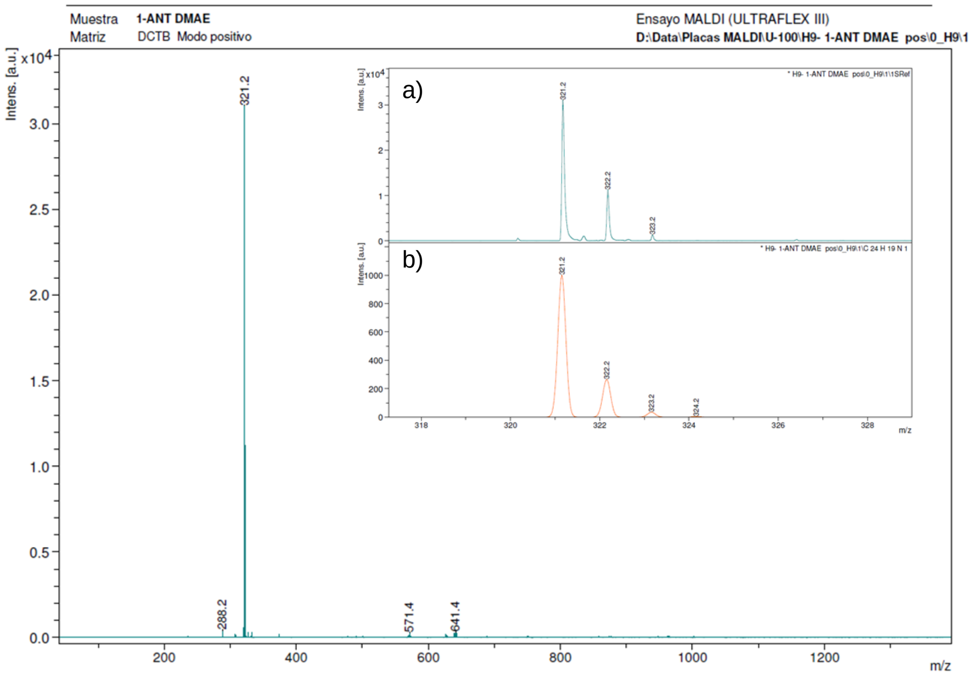

Figure S3.21. MALDI-TOF mass spectrum (DCTB matrix) of 1-(4-*N*,*N*-dimethylaminophenyl)-ethynylanthracene **4**. Inset: a) Isotopic distribution of the MALDI-TOF peaks between 318 and 328 m/z; b) calculated isotopic pattern for 1-(4-*N*,*N*-dimethylaminophenyl)-ethynylanthracene **4**.

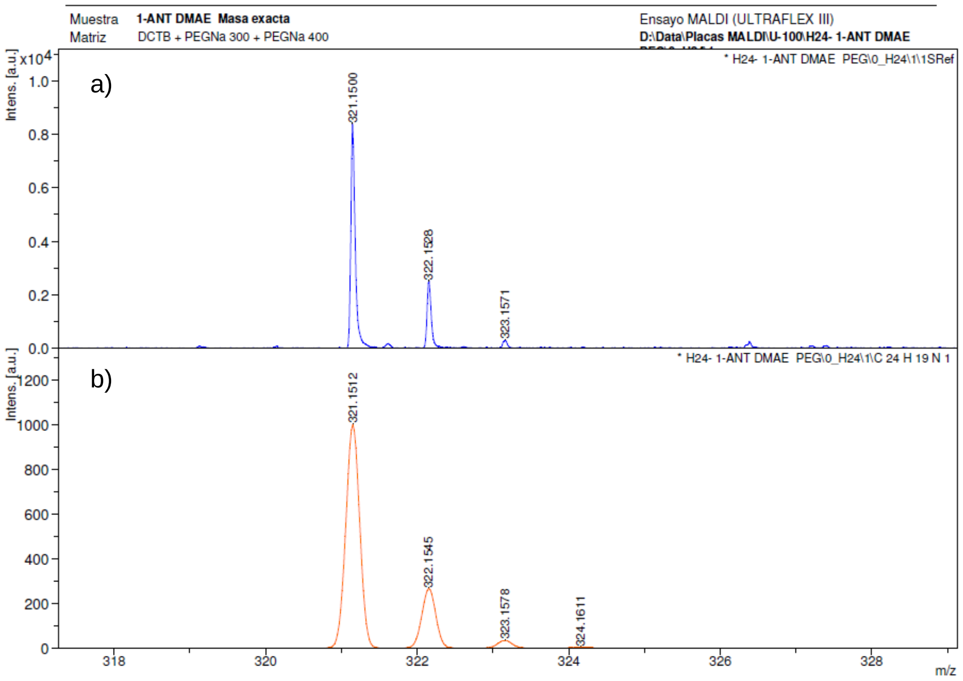

Figure S3.22. a) HRLSI-MS spectrum of 1-(4-*N*,*N*-dimethylaminophenyl)-ethynylanthracene **4**; b) calculated isotopic pattern for 1-(4-*N*,*N*-dimethylaminophenyl)-ethynylanthracene **4**.

Synthesis and characterization of anthryl−extTCNQ−DMA 5.

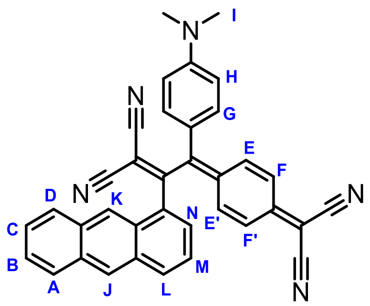

Compound **4** (20 mg, 0.062 mmol) was loaded in a 25 mL round-bottomed flask and dissolved in THF (3 mL). Then TCNQ (19 mg, 0.093 mmol) was added and the mixture was stirred for 16 h. The solvent was removed under reduced pressure and the mixture was subjected to column chromatography (SiO_2 gel, eluent = $CHCl_3$:EtOAc 99:1 v/v), where an intense grey band was collected. The resulting crude product was dissolved in the minimal amount of DCM and precipitated with *n*-hexane. The product was filtrated and washed with some more *n*-hexane, collected and dried under vacuum to yield anthryl−*ext*TCNQ−DMA **5** (13.6 mg, 42%) as a dark brown solid.

^{1}H-NMR (500 MHz, $CDCl_3$): δ 8.54 (s, 1H, H_K), 8.50 (s, 1H, H_J), 8.17 (d, J = 8.5 Hz, 1H, H_L), 8.06 – 8.01 (m, 1H, H_A), 8.01 – 7.95 (m, 1H, H_D), 7.64 (d, J = 6.5 Hz, 1H, H_N), 7.60 – 7.53 (m, 2H, H_{B+C}), 7.47 (dd, J = 8.4, 7.1 Hz, 1H, H_M), 7.37 (dd, *J* = 9.5, 1.9 Hz, 1H, $1xH_{E/E'/F/F'}$), 7.34 (dd, *J* = 9.6, 1.9 Hz, 1H, $1xH_{E/E'/F/F'}$), 7.31 (d, *J* = 9.0 Hz, 2H, H_G), 7.24 (dd, J = 9.5, 1.9 Hz, 1H, $1xH_{E/E'/F/F'}$), 7.15 (dd, J = 9.5, 1.9 Hz, 1H, $1xH_{E/E'/F/F'}$), 6.63 (d, J = 9.1 Hz, 2H, H_H), 3.07 (s, 6H, H_I); **^{13}C-NMR** (126 MHz, $CDCl_3$) δ (ppm) = 172.2, 153.9, 153.3, 152.9, 136.7, 135.1, 134.7, 134.4, 134.3, 132.8, 132.2, 131.6, 131.3, 128.7, 128.7, 128.2, 127.3, 127.3, 127.0, 125.7, 125.5, 125.1, 124.0, 123.4, 114.7, 114.7, 113.2, 112.7, 112.4, 91.6, 73.0, 40.2; **APCI** (positive mode): *m/z* 525.1947−529.2205 $[M+H]^{•+}$; **HRLSI-MS**: Calculated for $C_{36}H_{24}N_5$: 526.2026; Found: 526.2016; **UV/vis** ($CHCl_3$): λ_{max} (nm) (log ε) = 720 (4.38), 454 (4.24).

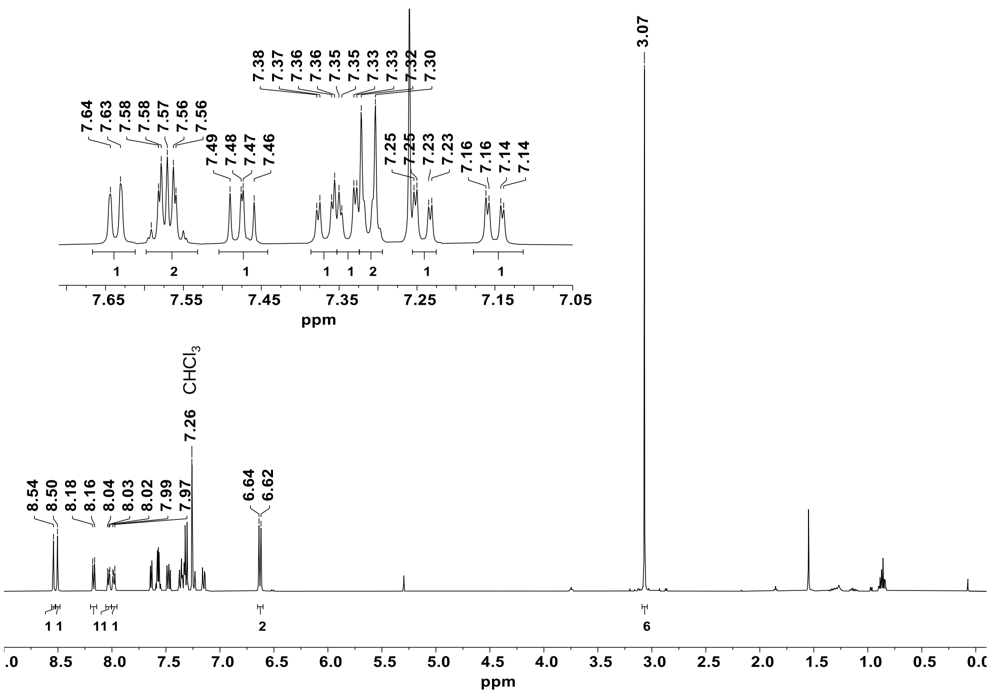

Figure S3.23. ^{1}H-NMR spectrum of anthryl−*ext*TCNQ−DMA **5** in $CDCl_3$.

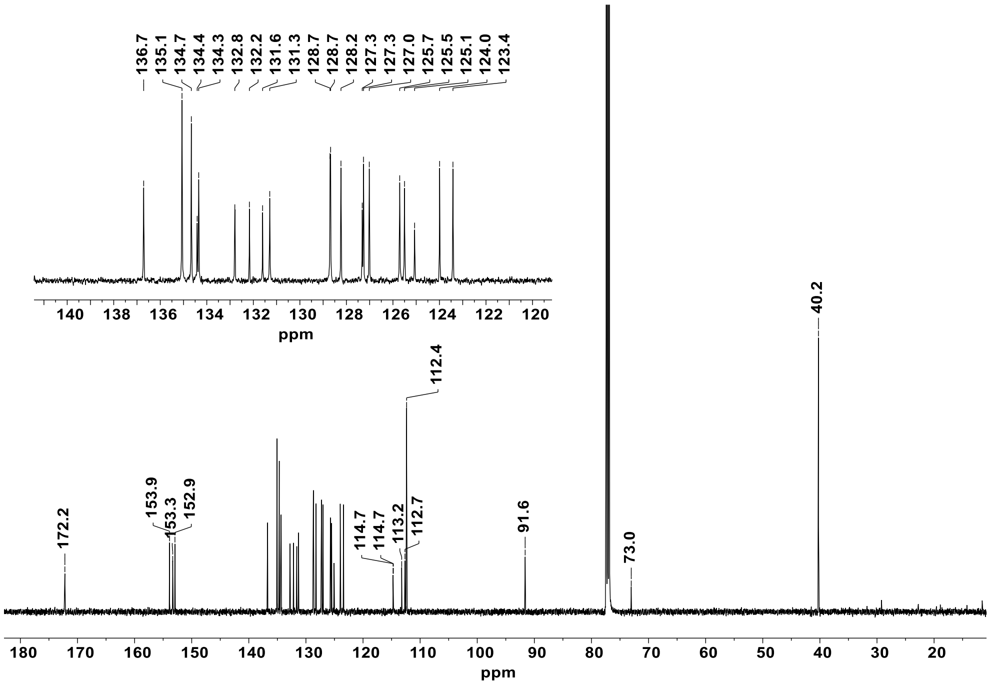

Figure S3.24. ^{13}C-NMR spectrum of anthryl−*ext*TCNQ−DMA **5** in $CDCl_3$.

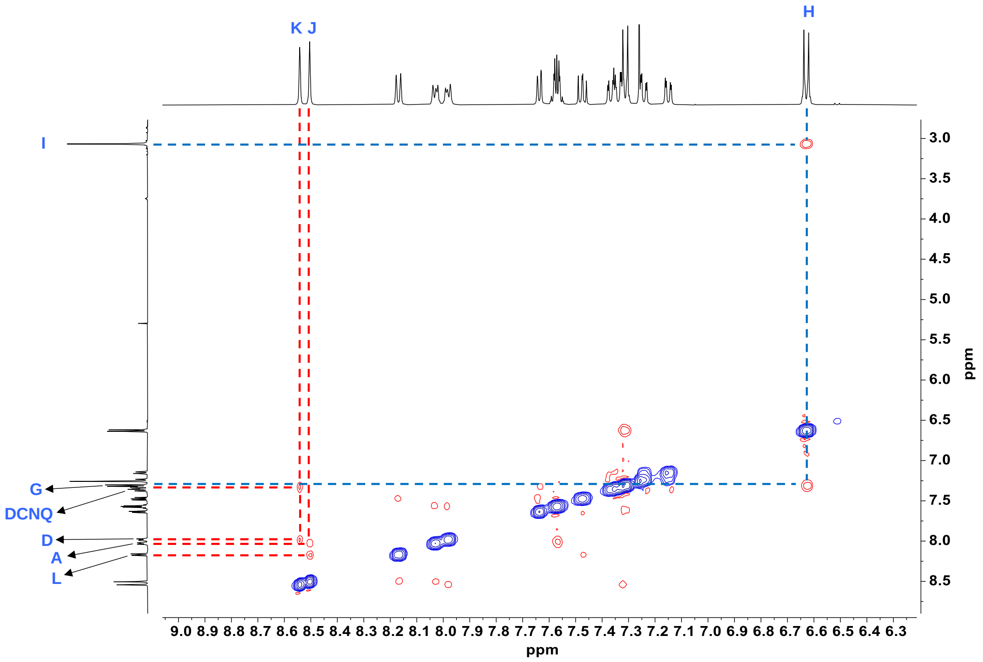

Figure S3.25. H-H NOESY NMR spectrum of anthryl−*ext*TCNQ−DMA **5** in $CDCl_3$.

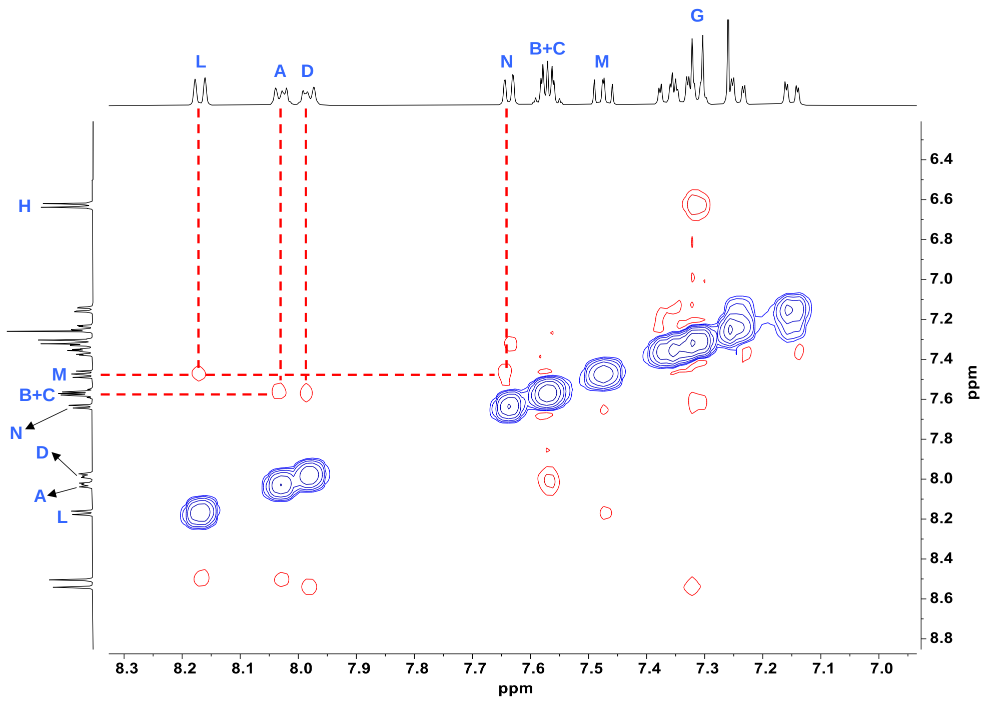

Figure S3.26. Portion of the H-H NOESY NMR spectrum of anthryl–*ext*TCNQ–DMA **5** in $CDCl_3$.

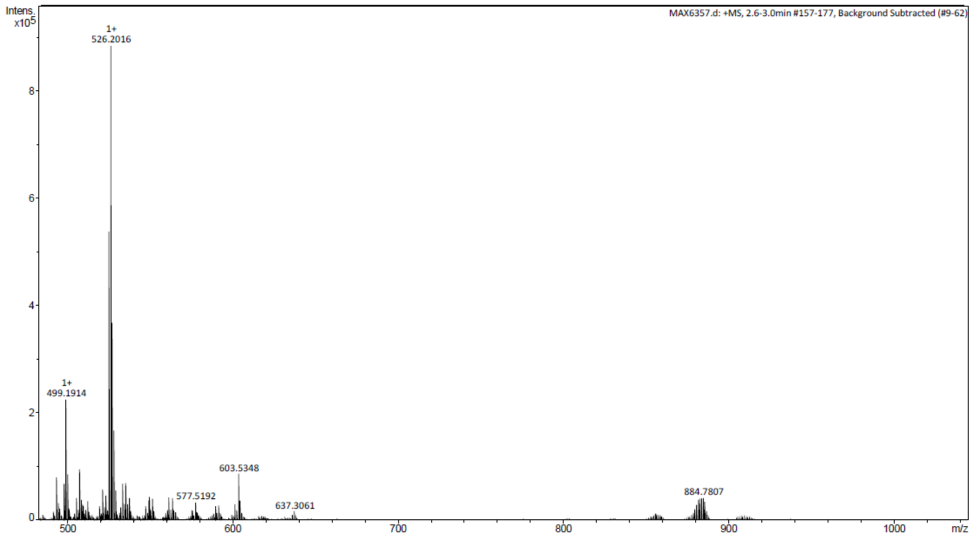

Figure S3.27. APCI (positive mode) mass spectrum of anthryl–*ext*TCNQ–DMA **5**.

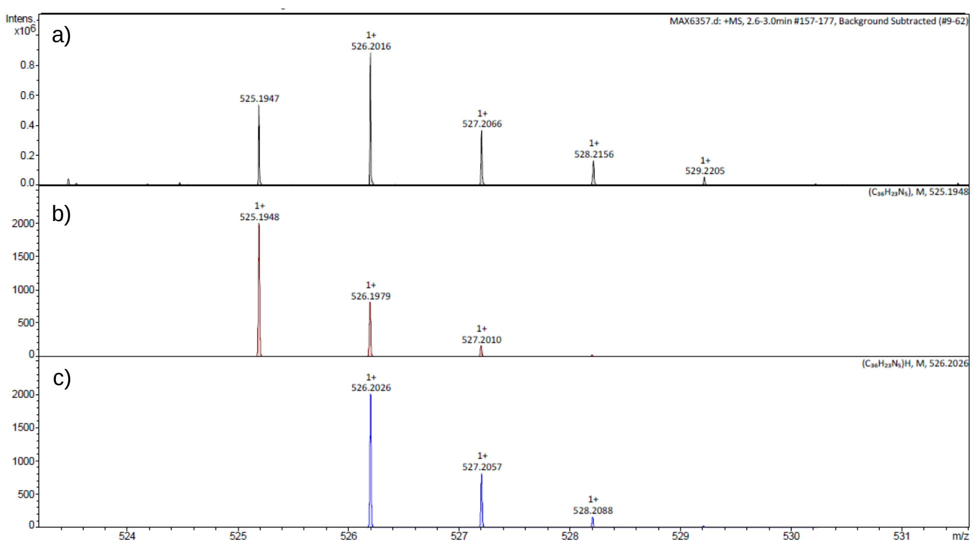

Figure S3.28. a) HRLSI-MS spectrum of anthryl–*ext*TCNQ–DMA **5**; b) calculated isotopic pattern for anthryl–*ext*TCNQ–DMA **5**; c) calculated isotopic pattern for anthryl–*ext*TCNQ–DMA [**5**+H].

Table S3.2. Selected crystallographic data for anthryl–*ext*TCNQ–DMA **5**.

**Chemical formula**	$C_{36}H_{23}N_5O_{1.83}$	
**Formula weight**	554.92 g/mol	
**Temperature**	296(2) K	
**Wavelength**	0.71073 Å	
**Crystal size**	0.012 × 0.023 × 0.190 mm	
**Crystal habit**	dark green-purple plate	
**Crystal system**	monoclinic	
**Space group**	P 1 21/n 1	
**Unit cell dimensions**	a = 11.040(3) Å	α = 90°
	b = 18.045(4) Å	β = 90.986(6)°
	c = 16.084(4) Å	γ = 90°
**Volume**	3203.7(12) Å^3	
**Z**	4	
**Density (calculated)**	1.150 g/cm^3	
**Absorption coefficient**	0.073 mm^{-1}	

**F(000)**	1155
**Theta range for data collection**	1.70 to 20.82°
**Index ranges**	–11<=h<=10, –18<=k<=18, –16<=l<=16
**Reflections collected**	26644
**Independent reflections**	3337 [R(int) = 0.1760]
**Coverage of independent reflections**	99.6%
**Absorption correction**	Multi-Scan
**Max. and min. transmission**	0.9990 and 0.9860
**Structure solution technique**	direct methods
**Structure solution program**	XT, VERSION 2018/2
**Refinement method**	Full-matrix least-squares on F^2
**Refinement program**	SHELXL-2019/1 (Sheldrick, 2019)
**Function minimized**	Σ w(F_o^2 - F_c^2)^2
**Data / restraints / parameters**	3337 / 324 / 392
**Goodness-of-fit on F^2**	1.095
**Final R indices**	1452 data; I>2σ(I) — R1 = 0.1674, wR2 = 0.4209
	all data — R1 = 0.2945, wR2 = 0.5242
**Weighting scheme**	w=1/[σ^2(F_o^2)+(0.2700P)^2+19.7000P]<br>where P=(F_o^2+2F_c^2)/3
**Largest diff. peak and hole**	1.369 and -0.569 eÅ^{-3}
**R.M.S. deviation from mean**	0.203 eÅ^{-3}

Synthesis and characterization of 4-((10-mesitylanthracen-9-yl)ethynyl)-N,N-dimethylaniline 6

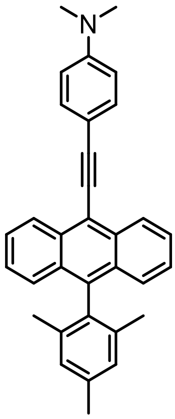

9-Bromo-10-mesitylanthracene (60 mg, 0.16 mmol) and $Pd(PPh_3)_4$ (9.2 mg, 0.008 mmol, 5 mol%) were added to a dried 25 mL Schlenk tube and subjected to three cycles vacuum and argon backfilling each. Then, under argon, dry DMF (4 mL) and NEt_3 (2 mL) were added, and the mixture was stirred. Then, sodium ascorbate (1.9 mg, 0.0096 mmol, 6 mol%) was added to the reaction mixture and it was stirred until dissolved. After this $CuSO_4{\cdot}5H_2O$ (0.8 mg, 0.0032 mmol, 2 mol%) was added. Finally, 4-ethynyl-*N,N*-dimethylaniline (30.2 mg, 0.208 mmol) was dissolved into dried DMF (1 mL) under argon and the solution was added to the reaction mixture dropwise. Then, the mixture was stirred at 80 ºC for 16 h. The reaction mixture was then allowed to reach r.t. and the crude was dissolved in EtOAc (20 mL) and washed with NH_4Cl (sat. sol. 15 mL), H_2O (2 × 10 mL) and brine (10 mL). After this, the organic layer was dried with anhydrous Na_2SO_4. The solution was filtered and the solvent was removed under reduced pressure. The solid was subjected to column chromatography (SiO_2 gel, 1:1 $CHCl_3$/heptane) where an intense yellow, fluorescent band was collected. The solvents were evaporated under reduced pressure. The resulting solid was dissolved in the minimum amount of CH_2Cl_2 and MeOH was added to the mixture. After evaporating the CH_2Cl_2, the solid was filtered and washed with some more MeOH, collected and dried in vacuum to yield **6** (54,7 mg, 78%) as a yellow solid.

^{1}H-NMR (500 MHz, $CDCl_3$): δ 8.76 (d, J = 8.7 Hz, 2H), 7.67 (d, J = 9.0 Hz, 2H), 7.61 – 7.52 (m, 2H), 7.49 (d, J = 8.6 Hz, 2H), 7.36 (ddd, J = 8.8, 6.4, 1.3 Hz, 2H), 7.09 (s, 2H), 6.78 (d, J = 9.0 Hz, 2H), 3.06 (s, 6H), 2.46 (s, 3H), 1.72 (s, 6H); **^{13}C-NMR** (126 MHz, $CDCl_3$) δ (ppm) = 150.4, 137.7, 137.4, 136.4, 134.6, 132.9, 132.4, 129.7, 128.4, 127.6,

126.5, 126.2, 125.9, 118.2, 112.2, 110.8, 102.5, 84.7, 40.5, 21.4, 20.1; **MALDI-TOF** (DCTB, positive mode): *m/z* 439.2278−441.2360 $[M]^{\bullet+}$; **HRLSI-MS**: Calculated for $C_{33}H_{29}N_1$: 439.2295; Found: 439.2278; **UV/vis** ($CHCl_3$): λ_{max} (nm) (log ε) = 446 (sh), 426 (4.39).

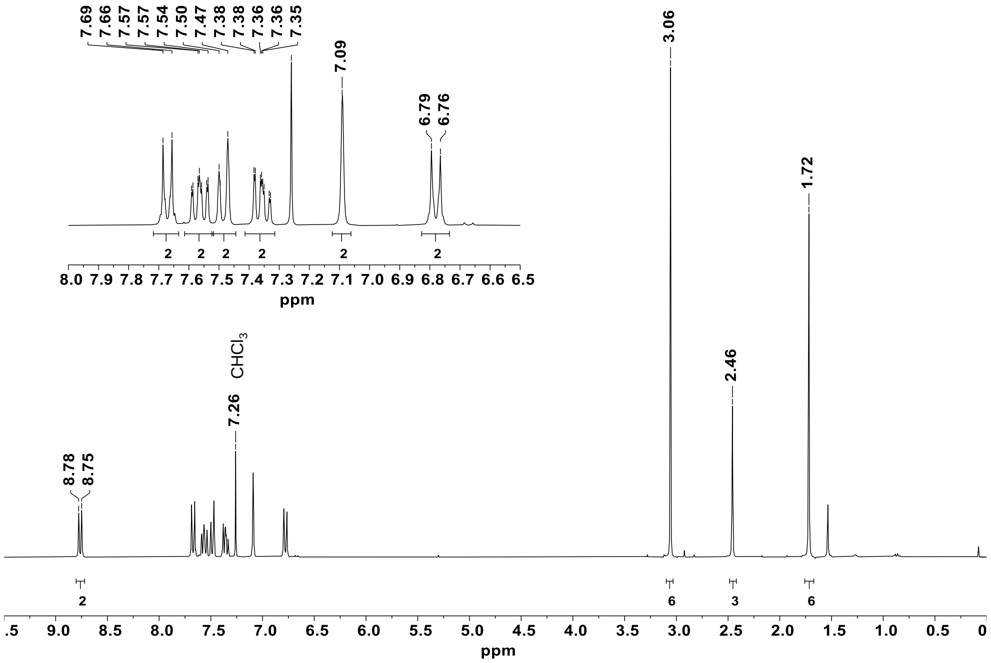

Figure S3.29. ^{1}H-NMR spectrum of 4-((10-mesitylanthracen-9-yl)ethynyl)-DMA **6** in $CDCl_3$.

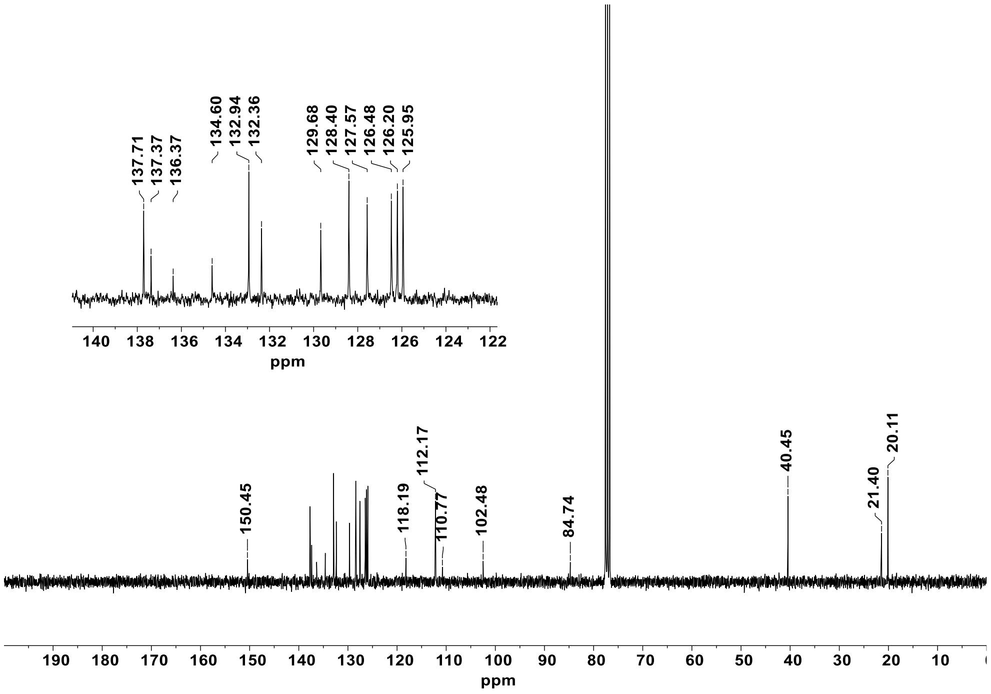

Figure S3.30. ^{13}C-NMR spectrum of 4-((10-mesitylanthracen-9-yl)ethynyl)-DMA **6** in $CDCl_3$.

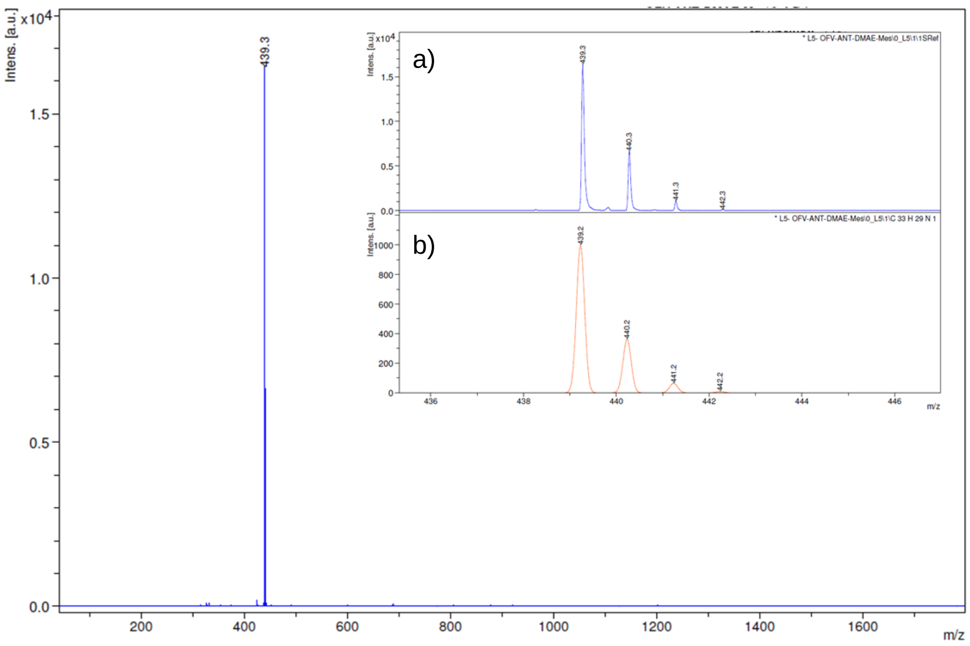

Figure S3.31. MALDI-TOF mass spectrum (DCTB matrix) of 4-((10-mesitylanthracen-9-yl)ethynyl)-DMA **6**. Inset: a) Isotopic distribution of the MALDI peaks between 436 and 446 m/z; b) calculated isotopic pattern for 4-((10-mesitylanthracen-9-yl)ethynyl)-DMA **6**.

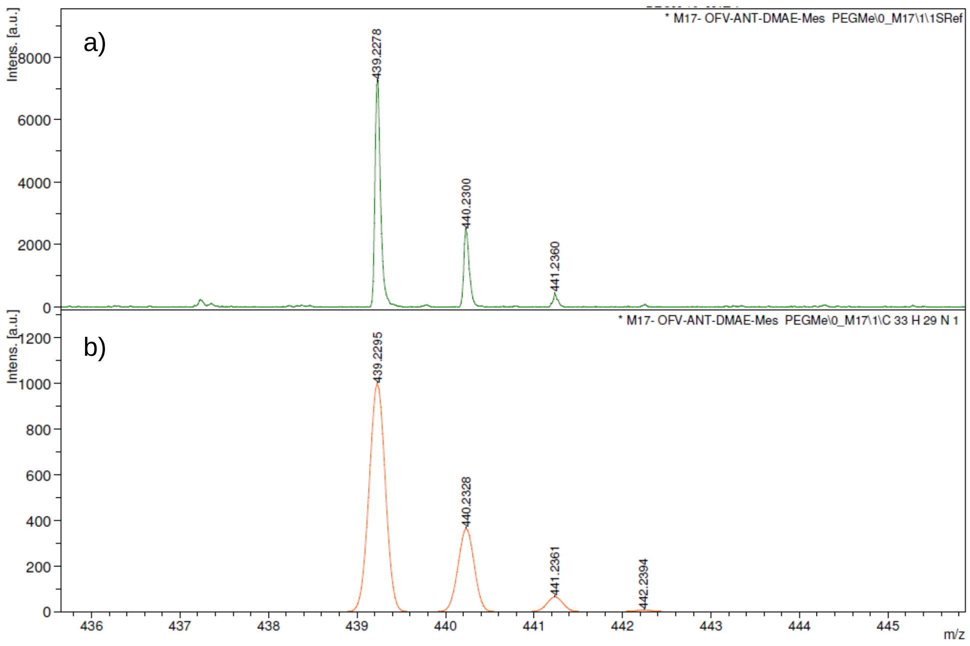

Figure S3.32. a) HRLSI-MS spectrum of 4-((10-mesitylanthracen-9-yl)ethynyl)-DMA **6**; b) calculated isotopic pattern for 4-((10-mesitylanthracen-9-yl)ethynyl)-DMA **6**.

Synthesis and characterization of anthryl−extTCNQ−DMA 7

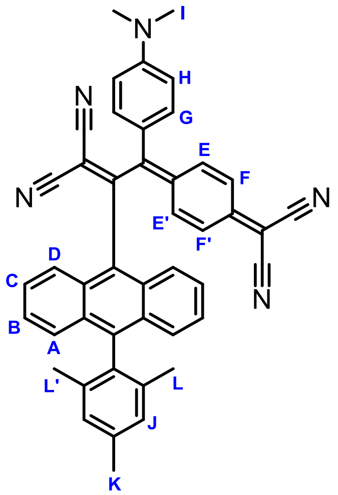

4-((10-mesitylanthracen-9-yl)ethynyl)-DMA **6** (22 mg, 0.034 mmol) was loaded in a 25 mL round-bottomed flask and dissolved in THF (5 mL). Then TCNQ (30.6 mg, 0.15 mmol, 4.4 eq.) was added and the mixture was stirred at 60 ºC for 16 h. The solvent was removed under reduced pressure and the mixture was subjected to column chromatography (SiO_2 gel, CH_2Cl_2:EtOAc 5%), where a black band was collected. The solvents were removed under reduced pressure and the crude product was subjected to size exclusion chromatography (BioBeads, $CHCl_3$). The solvent was then removed under reduced pressure and the resulting crude product was suspended in *n*-hexane, sonicated, filtered and washed with some more *n*-hexane, collected and dried in vacuum to yield anthryl−*ext*TCNQ−DMA **7** (23.1 mg, 72%) as a brown-black solid.

^{1}H-NMR (300 MHz, $CDCl_3$): δ 8.13 (d, J = 8.9 Hz, 2H, H_A), 7.65 – 7.57 (m, 2H, H_B), 7.55 (d, *J* = 8.7 Hz, 2H, H_D), 7.46 – 7.29 (m, 4H, H_G + H_C), 7.20 – 7.05 (m, 5H, H_J + 3×$H_{E/E'/F/F'}$), 6.83 (s (broad), 1H, 1×$H_{E/E'/F/F'}$), 6.65 (d, J = 8.7 Hz, 2H, H_H), 3.10 (s, 6H, H_I), 2.45 (s, 3H), 1.74 (s, 3H), 1.51 (s, 3H); **^{13}C-NMR** (75 MHz, $CDCl_3$) δ (ppm) = 169.8, 154.0, 153.2, 153.1, 143.4, 138.1, 137.7, 137.5, 137.2, 136.7, 135.8, 134.1, 133.7, 130.5, 130.3, 129.7, 129.0, 128.7, 128.6, 127.7, 126.3, 124.2, 114.6, 114.2, 112.6, 112.3, 95.6, 73.7, 40.3, 21.4, 20.1, 19.8; **MALDI-TOF** (DCTB, positive mode): *m/z* 643.2730−646.2953 $[M]^{•+}$; **HRLSI-MS**: Calculated for $C_{45}H_{33}N_5$: 643.2730; Found: 643.2730; **UV/vis** ($CHCl_3$): λ_{max} (nm) (log ε) = 787 (4.36), 584 (sh), 454 (sh), 416 (4.40), 396 (sh).

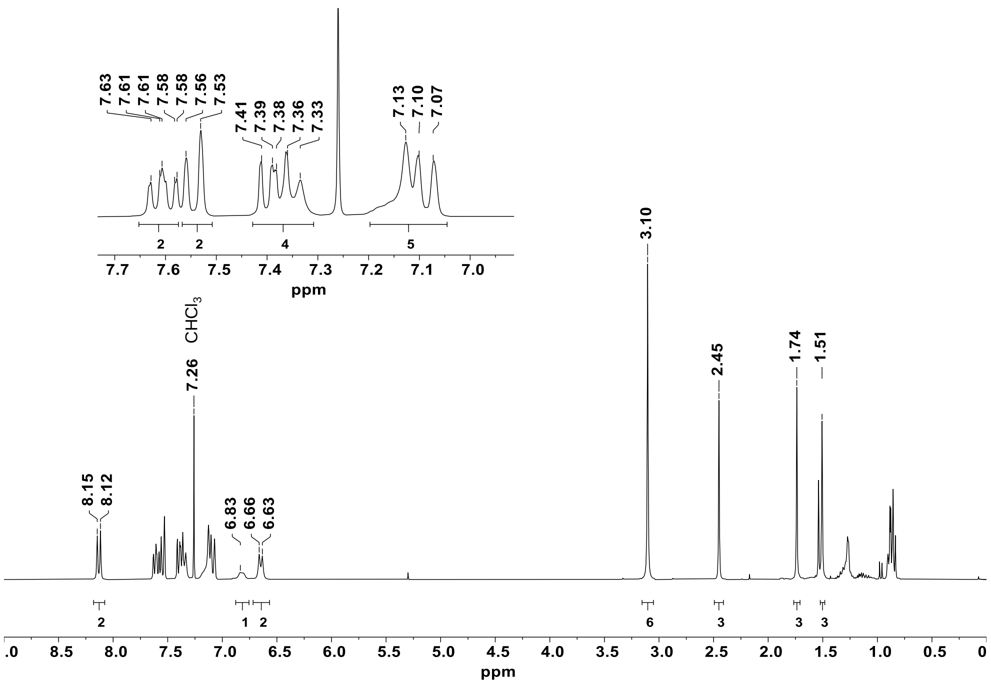

Figure S3.33. ^{1}H-NMR spectrum of anthryl−*ext*TCNQ−DMA **7** in $CDCl_3$.

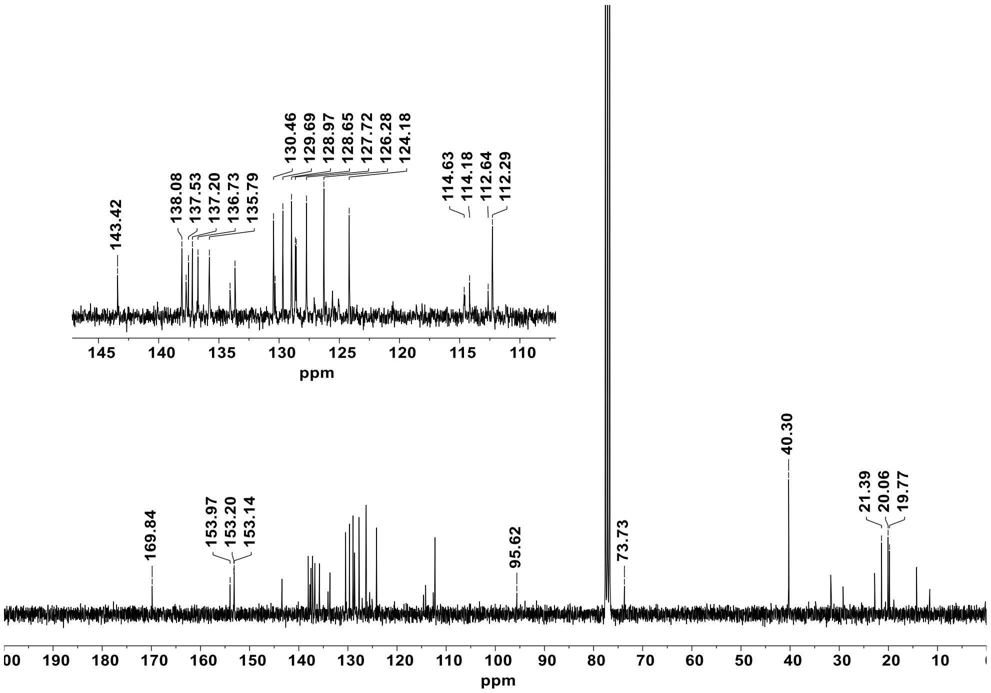

Figure S3.34. ^{13}C-NMR spectrum of anthryl−*ext*TCNQ−DMA **7** in $CDCl_3$.

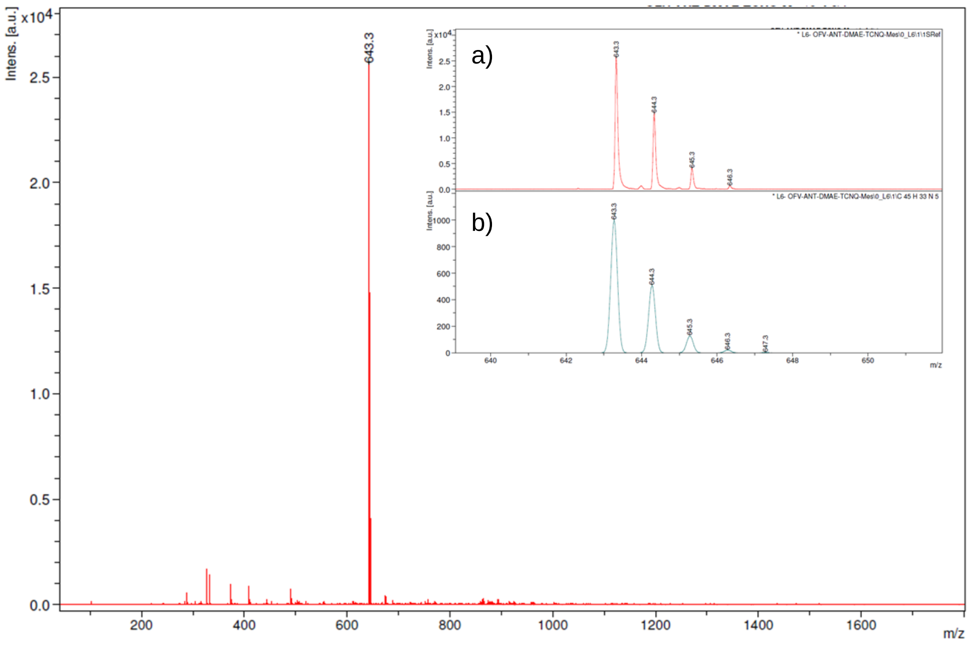

Figure S3.35. MALDI-TOF mass spectrum (DCTB matrix) of anthryl−*ext*TCNQ−DMA **7**. Inset: a) Isotopic distribution of the MALDI peaks between 640 and 651 m/z; b) calculated isotopic pattern for anthryl−*ext*TCNQ−DMA **7**.

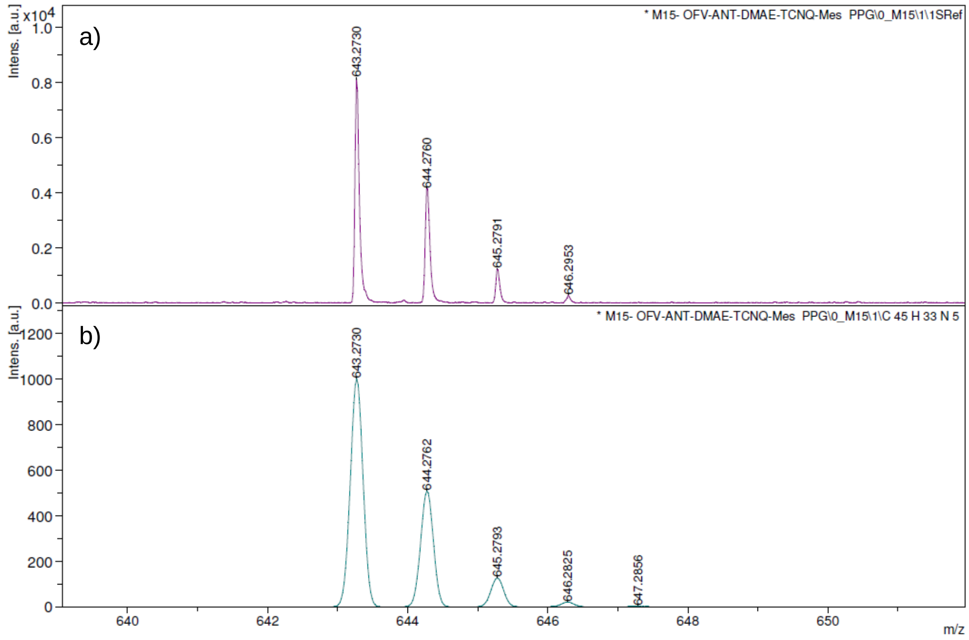

Figure S3.36. a) HRLSI-MS spectrum of anthryl−*ext*TCNQ−DMA **7**; b) calculated isotopic pattern for anthryl−*ext*TCNQ−DMA **7**.

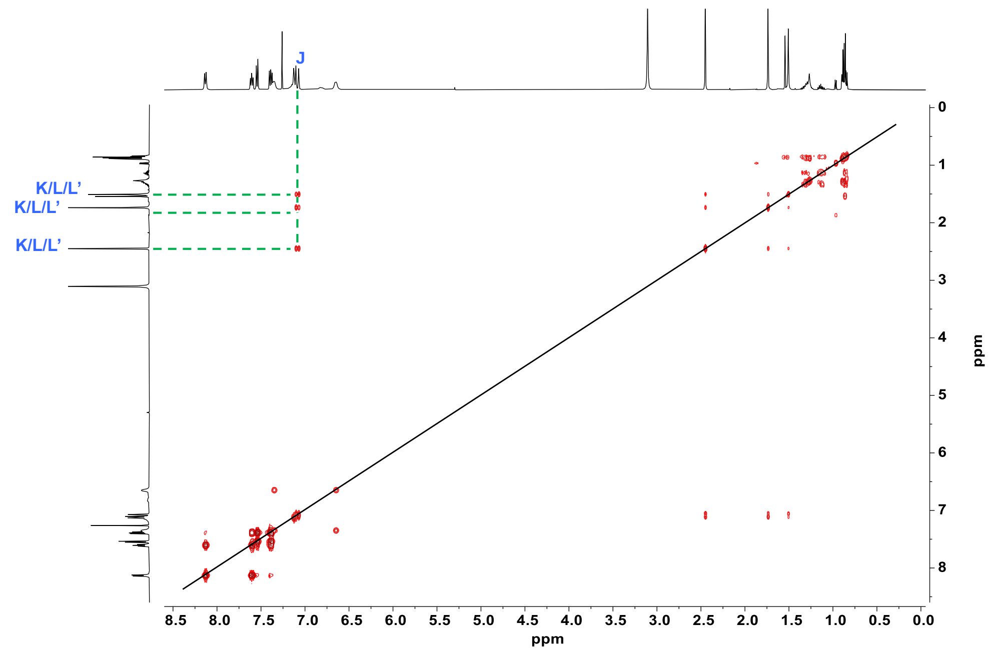

Figure S3.37. ^{1}H-COSY-NMR spectrum of anthryl−*ext*TCNQ−DMA **7** in $CDCl_3$ highlighting the correlations between the anthryl protons (red dashed lines), the protons of the DMA moiety (blue dashed lines) and the protons of the mesityl group (green dashed lines).

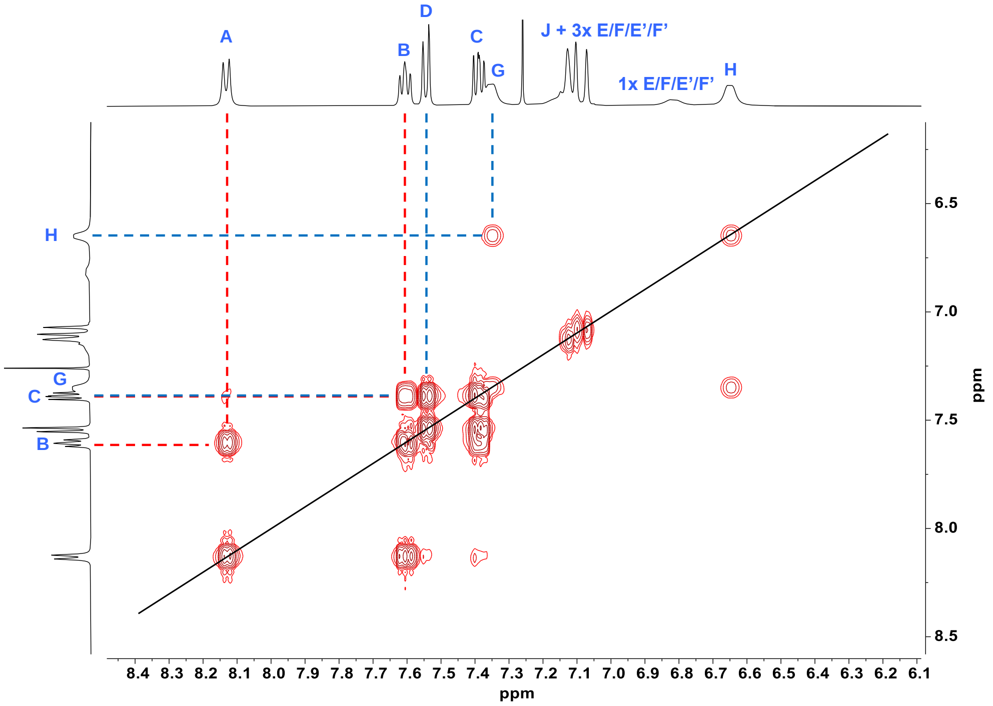

Figure S3.38. Portion of the ^{1}H-COSY-NMR spectrum of anthryl−*ext*TCNQ−DMA **7** in $CDCl_3$ highlighting the correlations between the anthryl protons (red dashed lines), the protons of the DMA moiety (blue dashed lines) and the protons of the mesityl group (green dashed lines).

Table S3.3. Selected crystallographic data for anthryl−*ext*TCNQ−DMA **7**.

**Empirical formula**	$C_{47}H_{35}Cl_6N_5$
**Formula weight**	882.50
**Temperature/K**	100.00(10)
**Crystal system**	monoclinic
**Space group**	I2/a
**a/Å**	15.2243(3)
**b/Å**	19.3926(5)
**c/Å**	29.7389(5)
**α/°**	90
**β/°**	90.842(2)
**γ/°**	90
**Volume/Å^3**	8779.1(3)
**Z**	8
**ρ_{calc}g/cm^3**	1.335
**μ/mm^{-1}**	3.878
**F(000)**	3632.0
**Crystal size/mm^3**	0.173 × 0.031 × 0.022
**Radiation**	Cu Kα (λ = 1.54184)
**2Θ range for data collection/°**	5.44 to 136.5
**Index ranges**	$-18 \le h \le 18$, $-21 \le k \le 23$, $-35 \le l \le 35$
**Reflections collected**	35306
**Independent reflections**	7995 [R_{int} = 0.0500, R_{sigma} = 0.0402]
**Data/restraints/parameters**	7995/0/528
**Goodness-of-fit on F^2**	1.051
**Final R indexes [I>=2σ (I)]**	R_1 = 0.0731, wR_2 = 0.2124
**Final R indexes [all data]**	R_1 = 0.0884, wR_2 = 0.2267
**Largest diff. peak/hole / e Å^{-3}**	1.56/–1.09

4. UV-vis absorption spectra of anthryl-fused–*ext*TCNQ–DMA 1, and anthryl−*ext*TCNQ−DMA 2, 5 and 7

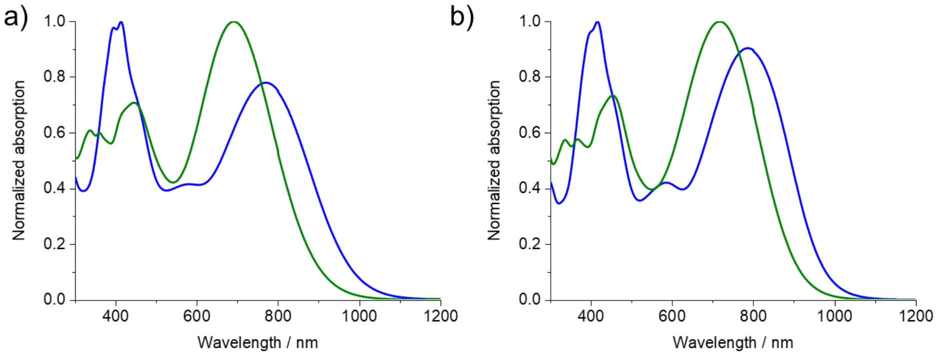

Figure S4.1. UV-vis absorption spectra of "expected" anthryl−*ext*TCNQ−DMA **5** (green line) and **7** (blue line) in a) THF and b) $CHCl_3$.

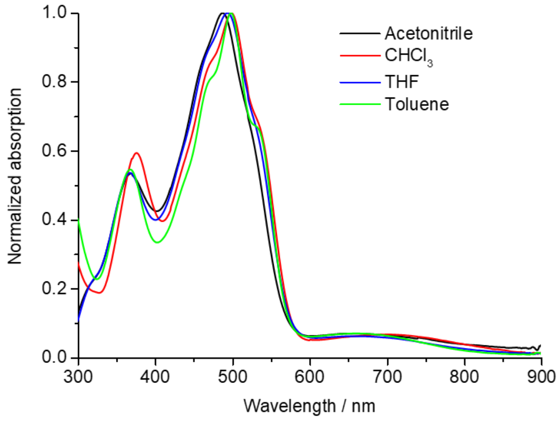

Figure S4.2. UV-vis absorption spectra of anthryl-fused–*ext*TCNQ–DMA **1** in acetonitrile (black line), chloroform (red line), THF (blue line), and toluene (green line).

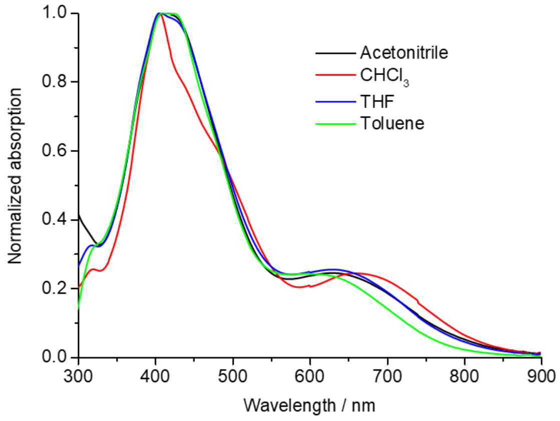

Figure S4.3. UV-vis absorption spectra of "unexpected" anthryl−*ext*TCNQ−DMA **2** in acetonitrile (black line), chloroform (red line), THF (blue line), and toluene (green line).

5. Electrochemical characterization of anthryl-fused−*ext*TCNQ−DMA 1 and anthryl−*ext*TCNQ−DMA 2

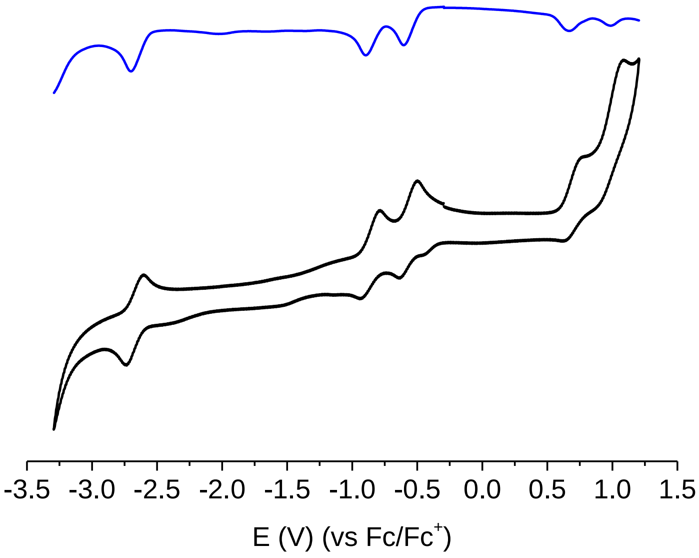

Figure S5.1. Cyclic voltammetry (bottom, black line) and differential pulse voltammetry (top, blue line) of anthryl−*ext*TCNQ−DMA **2** measured at a scan rate of 0.1 V s^{-1} in a 0.1 M solution of *n*-Bu_4NPF_6 in THF. Potentials are referred to $E_{1/2}$ of the Fc/Fc^+ redox couple.

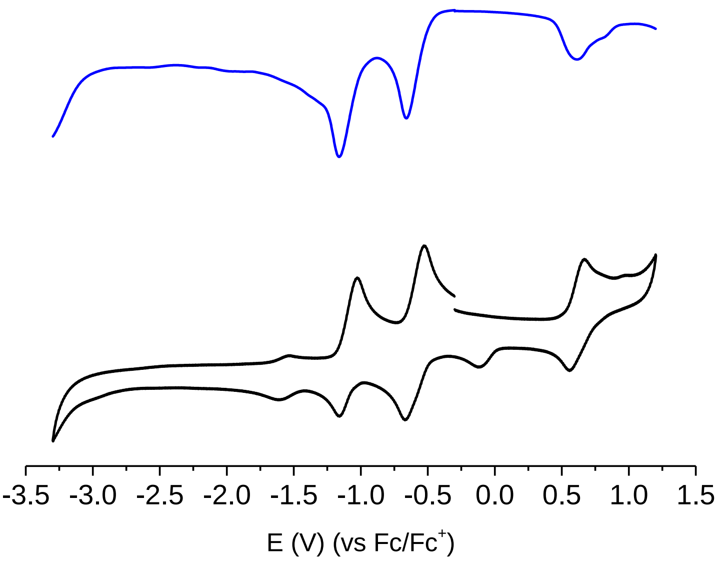

Figure S5.2. Cyclic voltammetry (bottom, black line) and differential pulse voltammetry (top, blue line) of anthryl-fused−*ext*TCNQ−DMA **1** measured at a scan rate of 0.1 V s^{-1} in a 0.1 M solution of *n*-Bu_4NPF_6 in THF. Potentials are referred to $E_{1/2}$ of the Fc/Fc^+ redox couple.

Table S5.1. Electrochemical data (*vs.* Fc/Fc$^+$) in THF of anthryl-fused−*ext*TCNQ−DMA **1** and anthryl−*ext*TCNQ−DMA **2** as determined by DPV.

Compound	$E_{1/2}^{ox,1}$ (V)	$E_{1/2}^{red,1}$ (V)	$E_{1/2}^{red,2}$ (V)	$E_{1/2}^{red,3}$ (V)
**2**	0.72	–0.60	–0.89	–2.7
**1**	0.67	–0.66	–1.16	

6. Quantum mechanical calculations studies

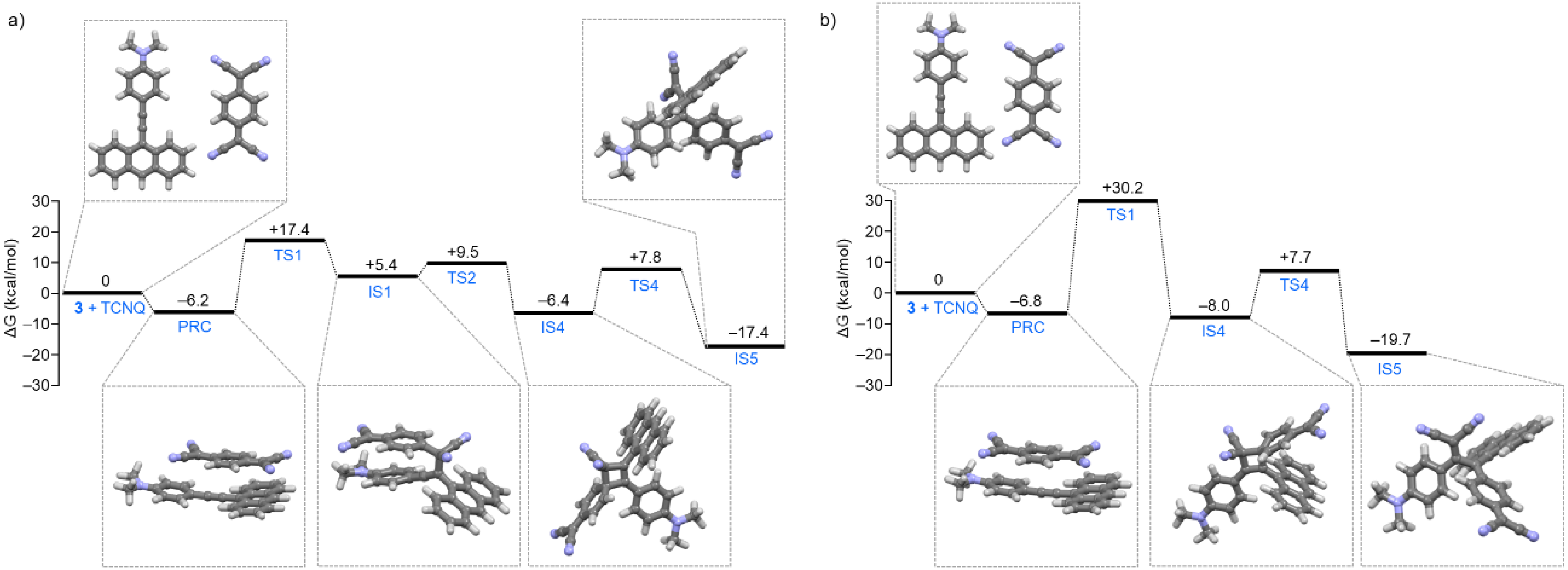

Figure S6.1. DFT-calculated reaction coordinates between 4-(anthracen-9-ylethynyl)-DMA **3** and TCNQ though an a) “on-pathway” and b) “off-pathway” [2+2] CA-RE reaction leading to the “expected” (*i.e.*, **2’**) and “unexpected” anthryl-fused−*ext*TCNQ−DMA (*i.e.*, **2**), respectively. The calculated structures of the two reactants, the pre-reaction complex (*i.e.*, PRC), and the intermediates (*i.e.*, IS) have been also represented and the respective energy levels included. The energy levels of the transition states (*i.e.*, TS) have also been included. Note that in the “off-pathway” mechanism, the PRC converts directly into the IS4 intermediate possibly due to the lack of an intermediate stabilized by a gain of aromatic as observed for the “on-pathway” mechanism (*i.e.*, IS1).

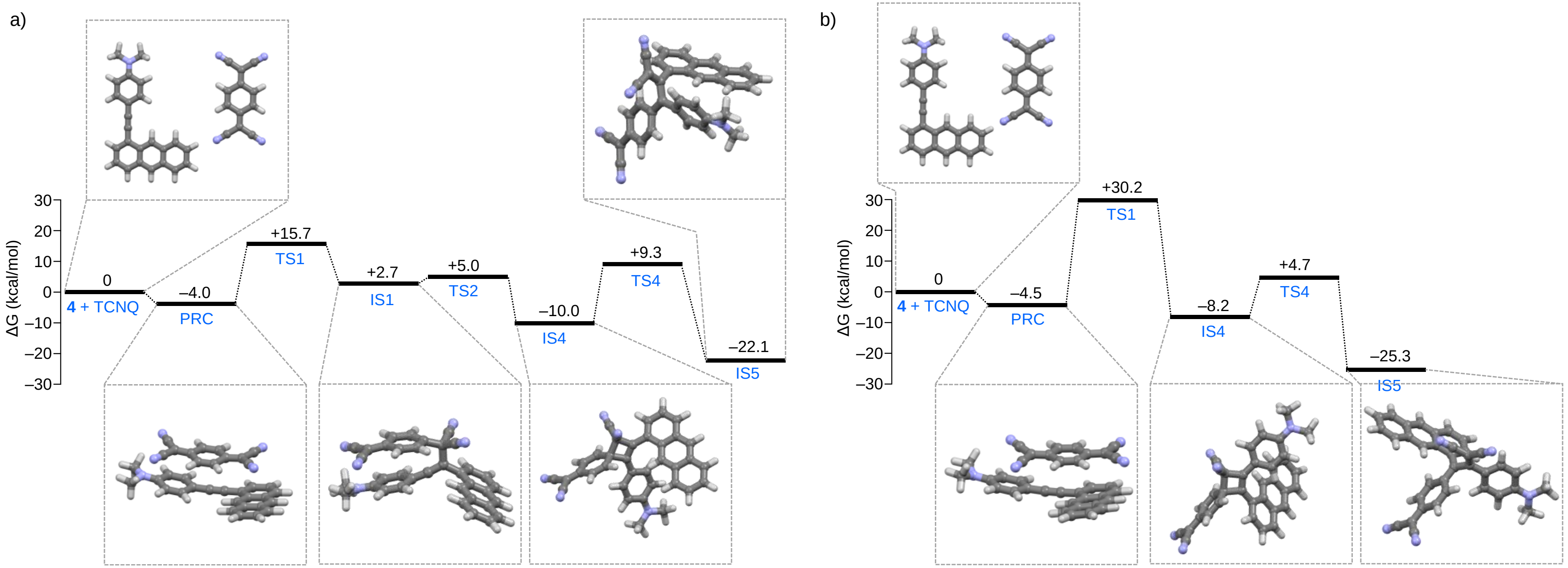

Figure S6.2. DFT-calculated reaction coordinates between 4-(anthracen-1-ylethynyl)-DMA **4** and TCNQ though an a) "on-pathway" and b) "off-pathway" [2+2] CA-RE reaction leading to "expected" anthryl-fused−*ext*TCNQ−DMA **5** and "unexpected" anthryl-fused−*ext*TCNQ−DMA **5'**, respectively. The calculated structures of the two reactants, the pre-reaction complex (*i.e.*, PRC), and the intermediates (*i.e.*, IS) have been also represented and the respective energy levels included. The energy levels of the transition states (*i.e.*, TS) have also been included. Note that in the "off-pathway" mechanism, the PRC converts directly into the IS4 intermediate possibly due to the lack of an intermediate stabilized by a gain of aromatic as observed for the "on-pathway" mechanism (*i.e.*, IS1).

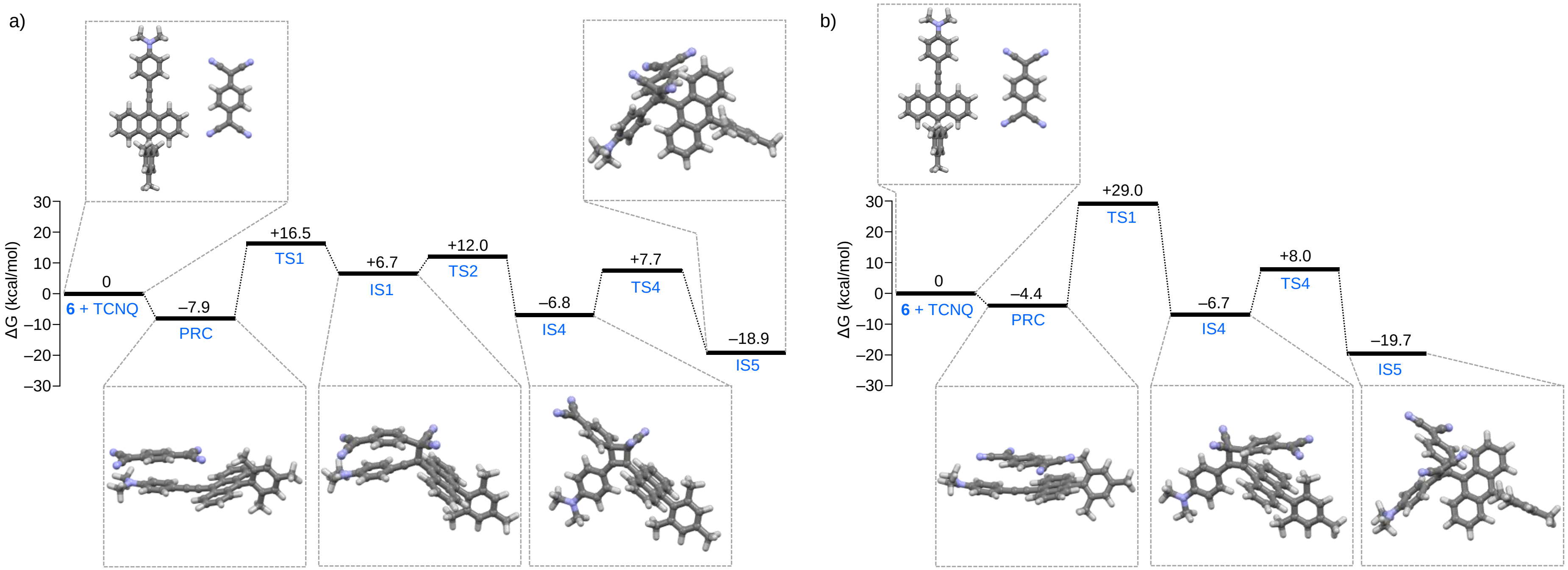

Figure S6.3. DFT-calculated reaction coordinates between 4-((10-mesitylanthracen-9-yl)ethynyl)-DMA **6** and TCNQ though an a) "on-pathway" and b) "off-pathway" [2+2] CA-RE reaction leading to "expected" anthryl-fused−*ext*TCNQ−DMA **7** and "unexpected" anthryl-fused−*ext*TCNQ−DMA **7'**, respectively. The calculated structures of the two reactants, the pre-reaction complex (*i.e.*, PRC), and the intermediates (*i.e.*, IS) have been also represented and the respective energy levels included. The energy levels of the transition states (*i.e.*, TS) have also been included. Note that in the "off-pathway" mechanism, the PRC converts directly into the IS4 intermediate possibly due to the lack of an intermediate stabilized by a gain of aromatic as observed for the "on-pathway" mechanism (*i.e.*, IS1).

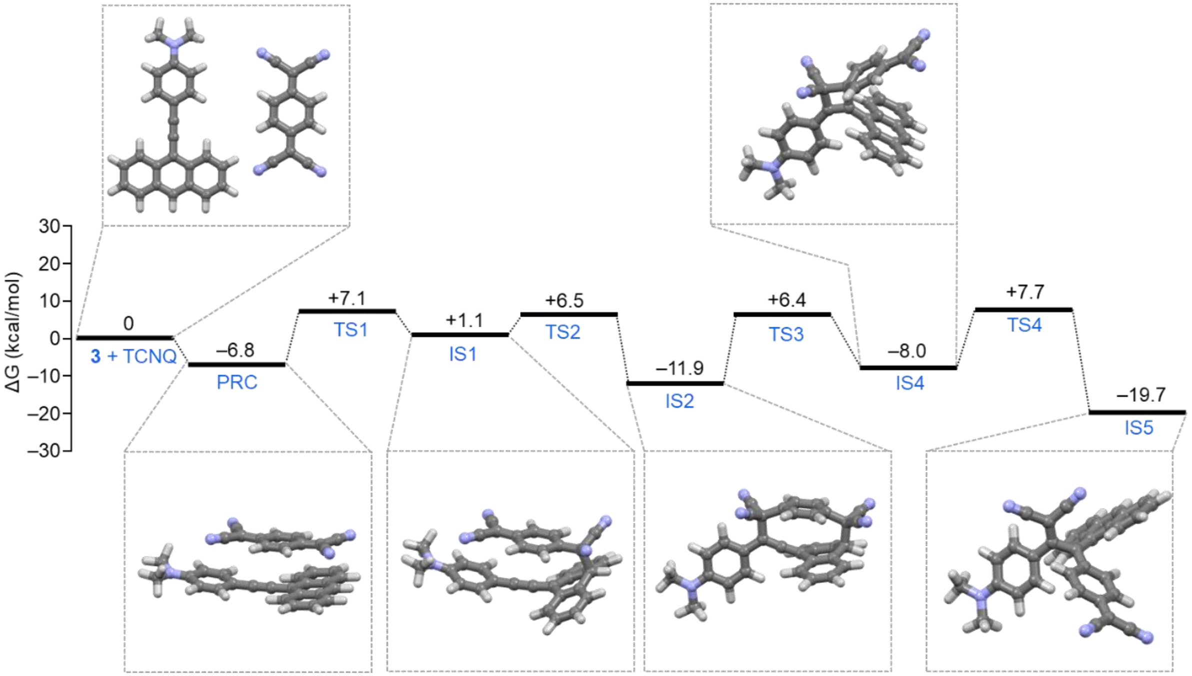

Figure S6.4. DFT-calculated reaction coordinates between 4-(anthracen-9-ylethynyl)-DMA **3** and TCNQ leading to the formation of "unexpected" anthryl-fused−*ext*TCNQ−DMA **2** through the formation of a [6+6] cycloadduct intermediate (*i.e.*, IS2) followed by a rearrangement. The calculated structures of the two reactants, the pre-reaction complex (*i.e.*, PRC), and the intermediates (*i.e.*, IS) have been also represented and the respective energy levels included. The energy levels of the transition states (*i.e.*, TS) have also been included.

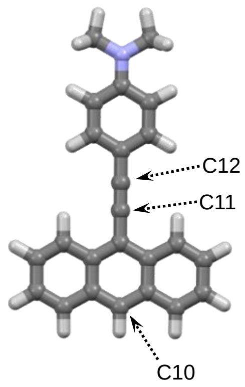

Atom site	f_i^+	f_i^-
C12	0.055	0.042
C11	0.014	0.059
C10	0.083	0.054

Figure S6.5. CFF for possible reactive sites of 4-(anthracen-9-ylethynyl)-DMA **3**.

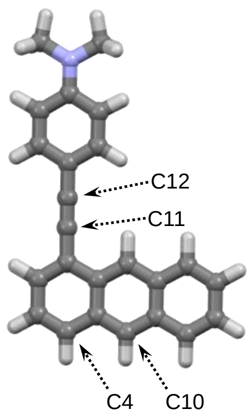

Atom site	f_i^+	f_i^-
C12	0.044	0.033
C11	0.010	0.071
C10	0.072	0.015
C4	0.064	0.035

Figure S6.6. CFF for possible reactive sites of 4-(anthracen-1-ylethynyl)-DMA **4**.

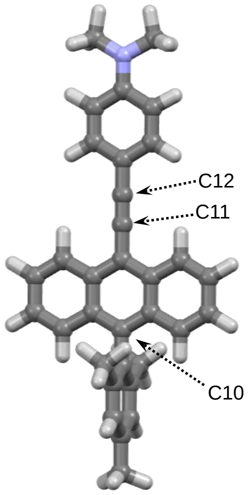

Atom site	f_i^+	f_i^-
C12	0.052	0.044
C11	0.013	0.056
C10	0.061	0.044

Figure S6.7. CFF for possible reactive sites of 4-((10-mesitylanthracen-9-yl)ethynyl)-DMA **6**.

7. Supporting Information References

1. Y. Zhao, D. G. Truhlar, *Theor. Chem. Acc.*, **2008**, *120*, 215–241.
2. N. Mardirossian, M. Head-Gordon, *Mol. Phys.*, **2017**, *115*, 2315–2372.
3. S. Pieniazek, F. R. Clemente, K. N. Houk, *Angew. Chem. Int. Ed.*, **2008**, *47*, 7746–7749.
4. T. A. Hamlin, B. J. Levandowski, A. K. Narsaria, K. N. Houk, F. M. Bickelhaupt, *Chem. Eur. J.*, **2019**, *25*, 6342–6348.
5. M. Frisch *et al.*, "Gaussian 16 Revision C. 01. 2016; Gaussian Inc.", Wallingford CT, vol. 29.
6. J. Tomasi, B. Mennucci, R. Cammi, *Chem. Rev.*, **2005**, *105*, 2999–3094.
7. W. Yang, W. J. Mortier, *J. Am. Chem. Soc.*, **1986**, *108*, 5708–5711.
8. L. Shubin, R. Chuying, L. Tian, *J. Phys. Chem. A*, **2014**, *118*, 3698–3704.
9. F. L. Hirshfeld, *Theor. Chim. Acta*, **1977**, *44*, 129–138.
10. B. Wang, C. Rong, P. K. Chattaraj, S. Liu, *Theor. Chem. Acc.*, **2019**, *138*, 124.
11. L. M. Mateo, L. Sagresti, Y. Luo, D. M. Guldi, T. Torres, G. Brancato, G. Bottari, *Chem. Eur. J.*, **2021**, *27*, 16049−16055.
12. L. Valenta, M. Mayländer, P. Kappeler, O. Blacque, T. Šolomek, S. Richert, M. Juríček, *Chem. Commun.*, **2022**, *58*, 3019–3022.
